# Residual Tensile Strength of CFRP Panels with Dual Stepped-Lap Repairs: Effects of Ligament Spacing, Patch Lay-Up, and Repair Arrangement

**DOI:** 10.3390/polym18212757

**Published:** 2026-11-05

**Authors:** Alihan Cambaz, Huseyin Enes Salman

**Affiliations:** Department of Mechanical Engineering, TOBB University of Economics and Technology, Ankara 06560, Türkiye; a.cambaz@etu.edu.tr

**Keywords:** stepped-lap composite repair, multi-site damage, residual tensile strength, cohesive zone modelling, finite element analysis

## Abstract

On-site repair of carbon fibre-reinforced polymer (CFRP) structures is widely employed when component removal and autoclave-based processing are impractical. However, existing repair guidelines provide limited guidance on the interaction between closely spaced repairs. The objective of this research is to investigate the coupled effects of ligament spacing, patch lay-up, and repair arrangement on the residual tensile strength of dual stepped-lap repaired composite panels. The methodology consists of a shell-based finite element model combining Hashin intralaminar damage and a mixed-mode bilinear cohesive zone model, which was validated against published experiments on damaged, stepped, and wet lay-up repaired laminates, predicting the measured strengths within 6.4%. Subsequently, fifteen repair configurations were analyzed, comprising four ligament spacings, three patch lay-up strategies, and two repair arrangements. Residual strength varied non-monotonically with ligament spacing for repairs arranged transverse to the loading direction. For the baseline patch, the strength retention factor increased from 0.57 for closely spaced repairs to a maximum of 0.85 at 120 mm, and decreased to 0.59 at 180 mm as the repairs approached the panel free edges. The compliant ±45° patch was the most sensitive to spacing (0.42–0.80), whereas the stiffness-matched patch varied only between 0.48 and 0.61. In contrast, in-line repairs showed limited sensitivity to spacing, retaining 0.72–0.77 of the intact strength. In conclusion, the results demonstrate that repair orientation relative to the principal load path governs the interaction between adjacent repairs more strongly than separation distance alone.

## 1. Introduction

Carbon fibre-reinforced polymers (CFRPs) have become a key material class in modern aerospace engineering, underpinning structural designs in both commercial transport and advanced military aircraft. In civil aviation, composite structural architectures range from modular, panelized skin assemblies to large-scale, monolithic co-cured barrel structures. These approaches are primarily aimed at achieving aerodynamic surface continuity while reducing the reliance on mechanical fasteners [1]. In military aircraft, CFRPs are similarly integral to structural design, owing not only to their high specific stiffness and strength but also to their contribution to radar cross-section reduction and low-observability requirements [2].

Despite their excellent in-plane tensile and compressive performance along the principal fibre directions, laminated composites exhibit comparatively poor matrix-dominated and through-thickness properties. This inherent anisotropy makes aerospace composite structures particularly susceptible to out-of-plane loading, including low-velocity impacts such as tool drops and hail in civil aviation, as well as higher-energy impact events associated with shrapnel and ballistic fragmentation in military applications [3,4]. Such loading can generate extensive internal delamination and fibre–matrix debonding, resulting in substantial reductions in residual structural capacity despite limited or barely detectable surface damage. For large-scale and highly integrated primary structures, complete component replacement is often impractical from both logistical and economic perspectives. Consequently, reliable bonded repair techniques capable of restoring structural integrity and load-carrying capability are of critical importance [5,6,7].

In aerospace maintenance, removing damaged composite panels for depot-level prepreg repairs, which typically require large-scale autoclave processing, can result in substantial aircraft downtime. This limitation has driven the widespread use of rapid, in-situ field repairs based on wet lay-up bonded repair techniques [8,9]. Such repairs are performed directly on the airframe using portable vacuum-bagging systems and localized heating blankets [10,11,12], with the objective of restoring structural integrity while maintaining the required aerodynamic surface continuity without autoclave-level consolidation. To promote efficient load transfer and mitigate the stress concentrations associated with mechanically fastened repairs, stepped-lap and scarf-bonded configurations are widely employed [13,14].

The mechanical response and failure progression of isolated stepped-lap repairs have been extensively investigated in recent years. Sonat et al. [11,12], for example, provided a comprehensive assessment of failure mechanisms in stepped-lap CFRP laminates, highlighting damage modes including interlaminar delamination and cohesive failure at the adhesive–adherend interface. Damghani et al. [15,16] investigated the optimization of stepped-joint geometries, examining the effects of step length and stiffness distribution to reduce the extent of pristine material removal while improving load transfer in highly loaded composite structures. To capture these failure mechanisms, contemporary numerical approaches commonly employ cohesive zone modeling (CZM) to describe damage initiation and subsequent degradation of the adhesive interface [7,17]. Accordingly, numerous coupon-level investigations have combined experimental testing with CZM-based numerical analyses to optimize stepped-lap repair parameters, including step overlap length, ply-drop ratio, and adhesive thickness [18,19,20,21]. However, operational damage and aircraft battle damage repair (ABDR) scenarios can involve multiple damage sites occurring in close proximity, resulting in multi-site damage (MSD) clusters [22]. The assessment and repair of such adjacent damage zones can be strongly influenced by the conservative provisions of structural repair manual (SRM) guidelines. When the separation between individual damage sites falls below the prescribed minimum repair distance, SRM procedures may require the repair boundaries to overlap, effectively combining the adjacent damage sites into a single repair region [23,24]. This approach can necessitate the removal of additional undamaged parent material to accommodate a larger stepped-lap or scarf repair configuration. Such a conventional merging strategy can introduce significant structural challenges.

**Figure 1:**
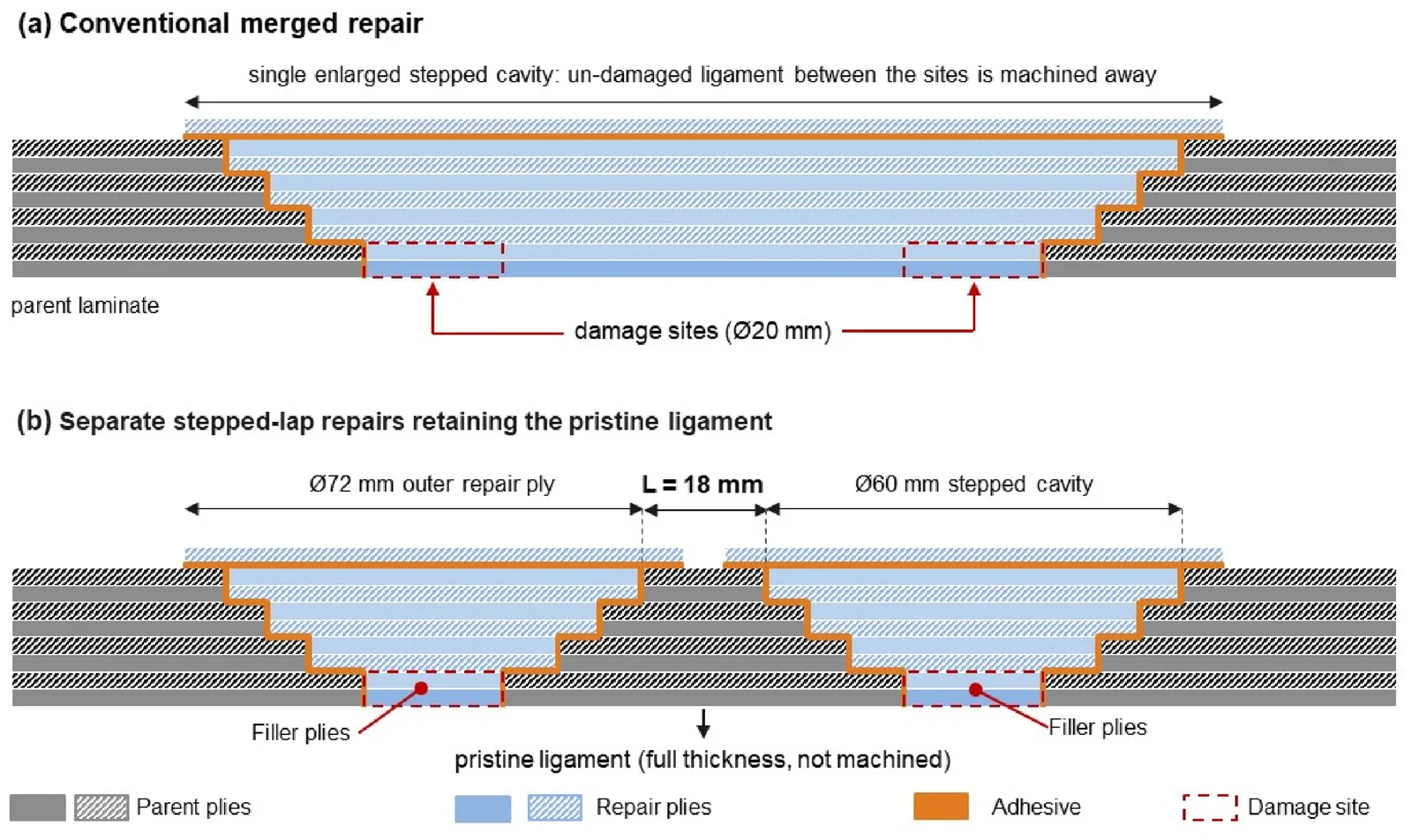
Schematic of the repair strategies for two closely spaced damage sites: (**a**) conventional merged repair, in which both sites are enclosed in a single, enlarged stepped cavity; (**b**) separate stepped-lap repairs retaining the pristine ligament between them, as investigated in the present study. The through-thickness scale is exaggerated for clarity.

When multiple damage sites are consolidated into a single large cutout, the resulting reduction in the effective load-bearing cross-section can substantially increase the structural demand on the remaining parent material and may compromise the ability of the repaired configuration to sustain limit load under an assumed bondline failure, as required for bonded joints whose failure would be catastrophic [25,26]. Accordingly, minimizing the overall repair footprint can help preserve the load-carrying capability of the parent structure while satisfying applicable airworthiness requirements [14,25,26] and several optimized geometries have been proposed with this objective [27,28,29,30]. In this context, treating adjacent damage sites as separate repair entities, with localized filler plies introduced where required, provides an alternative to unnecessary removal of pristine material and allows a greater portion of the original load path to be retained. This repair strategy is adopted and evaluated in the present work. The conventional merged repair and the separate-repair strategy considered here are compared schematically in Figure 1a,b.

While retaining the pristine ligament between closely spaced repairs supports compliance with the global limit-load requirement, it introduces a localized structural challenge arising from the interaction of the stress fields associated with adjacent repairs. The locally stiffened repair patches can perturb the surrounding load path and generate interacting stress concentrations within the intervening parent material. Kashfuddoja and Ramji [31] demonstrated using digital image correlation and finite element analysis that patch geometry strongly influences the strain concentration in the surrounding parent material, while Caminero et al. [32] similarly characterized localized strain fields around adhesively bonded repairs through full-field measurements. However, although the stress concentration associated with an individual bonded repair has been extensively investigated, the interaction between two closely spaced repairs and, critically, the residual load-carrying capability of the ligament between them remains insufficiently addressed in the open literature. For laminates with multiple open holes, Alharthi and Abdellah [33] showed that the interaction between neighboring cut-outs depends strongly on their alignment relative to the load; whether comparable trends hold for adjacent bonded repairs, whose locally stiffened patches alter the load path differently from open holes, has not been established.

A comprehensive assessment of this interaction also requires consideration beyond conventional coupon-scale configurations. One factor contributing to the conservatism of SRM-based repair provisions is the predominant reliance of bonded-joint and bonded-repair research on small-scale coupon specimens [5,7]. Although coupon tests remain essential for characterizing adhesive properties and fundamental failure mechanisms, their limited dimensions constrain transverse deformation and promote predominantly uniaxial load transfer. In aircraft-scale panels, however, the surrounding structure provides additional load-transfer paths, allowing far-field loads to bypass locally stiffened repair regions and redistribute through the adjacent structure. Consequently, the retained ligament may experience a more complex and multiaxial stress state than that captured in isolated coupon configurations. As demonstrated by panel-level investigations of repaired composite structures [34], structural-scale analyses are therefore required to capture load redistribution, load-bypass mechanisms, and the residual structural capability of closely spaced stepped-lap repairs under representative boundary conditions.

The stacking sequence of the wet lay-up repair patch further governs the local stiffness and, consequently, the load-transfer path through the repaired region [12,34,35]. When two repairs are located in close proximity, the stiffness mismatch between the composite patches and the parent laminate can intensify local stress concentrations and alter the load-transfer mechanism. This mismatch can increase interlaminar shear and peel stresses within the constrained ligament region [13,35]. Although the effect of patch lay-up has been extensively investigated for isolated repairs [13,18,35], its coupled influence with the minimum allowable ligament spacing in dual-site stepped-lap configurations remains insufficiently characterized, representing an important research gap in the assessment of closely spaced composite repairs.

To address this research gap, the present study investigates the interaction mechanisms of dual-site stepped-lap composite repairs at the panel level. In contrast to conventional coupon-scale investigations, the analysis captures load redistribution and load-bypass mechanisms within a large-scale structural configuration. Although aerospace skin panels may be subjected predominantly to in-plane shear loading, post-buckling deformation can alter the load path through the development of a diagonal tension field. Accordingly, uniaxial tensile loading is considered to provide a conservative and structurally relevant basis for assessing the ultimate response of the repaired panels. In addition, free boundary conditions are imposed along the transverse edges, allowing local load redistribution and stress concentrations to develop without transverse constraint and thereby providing a conservative assessment of the repair configuration. The coupled effects of repair proximity and patch stiffness are systematically investigated through 15 distinct configurations. For two repairs placed side by side transverse to the loading direction, four ligament spacing distances, ranging from closely interacting repairs (18 mm) to a nominally isolated configuration (180 mm), are examined in combination with three patch stacking sequences: a baseline reference lay-up, a stiffness-matched lay-up, and a highly compliant angle-ply lay-up ([±45°/±45°]). Three additional configurations with the two repairs arranged in line with the loading direction isolate the effect of repair orientation. The resulting parametric framework enables the influence of repair spacing and patch stiffness on the structural response of dual-site repairs to be evaluated systematically at the panel level. The findings provide a mechanics-based basis for assessing closely spaced multi-site composite repairs and offer guidance for the optimization of wet lay-up field-repair configurations and the evaluation of existing repair-spacing provisions in aerospace composite structures.

The novelty of the present study lies in three aspects. First, to the best of the authors’ knowledge, it provides the first panel-level assessment of two adjacent stepped-lap repairs that retain the pristine ligament between them, as an alternative to the conventional SRM practice of merging closely spaced damage sites into a single, larger repair. Second, ligament spacing, patch lay-up and repair arrangement are investigated in combination rather than individually, which reveals coupled effects that cannot be identified from isolated-repair or coupon-level studies: a non-monotonic dependence of residual strength on spacing for the transverse arrangement, governed by the transition from repair-to-repair to repair-to-free-edge interaction, and a much weaker dependence for the in-line arrangement. Third, the comparison of patch lay-ups shows that, in stepped-lap repairs, matching the ply orientation at each bonded overlap is more important than matching the global stiffness of the patch. Together, these results provide a mechanics-based basis for evaluating repair-spacing provisions for multi-site damage in composite structures.

The study is carried out in two stages. In the first stage (Section 2, Section 3.1 and Section 3.2), the finite element modelling approach, comprising the shell discretization, the Hashin intralaminar damage model and the mixed-mode cohesive interface, is validated against the damaged, stepped and wet lay-up repaired coupons of the reference experiments [12]. In the second stage (Section 3.3), the validated approach is applied to a 400 × 320 mm panel containing two stepped-lap repairs, arranged either transverse to or in line with the loading direction, and the interfacial damage topology, peak axial stress, failure load and strength retention factor are evaluated for each configuration. The main findings and their implications for repair design are summarized in Section 4.

## 2. Materials and Methods

In this study, finite element models are used to determine the mechanical behavior of the dual-repaired cases. For this purpose, the employed finite element modeling strategy is validated using a reference experimental study from the literature [12]. All the material properties, constitutive models, and damage evaluation criteria are explained in this section.

### 2.1. Validation of the Finite Element Modeling Approach

**Figure 2:**
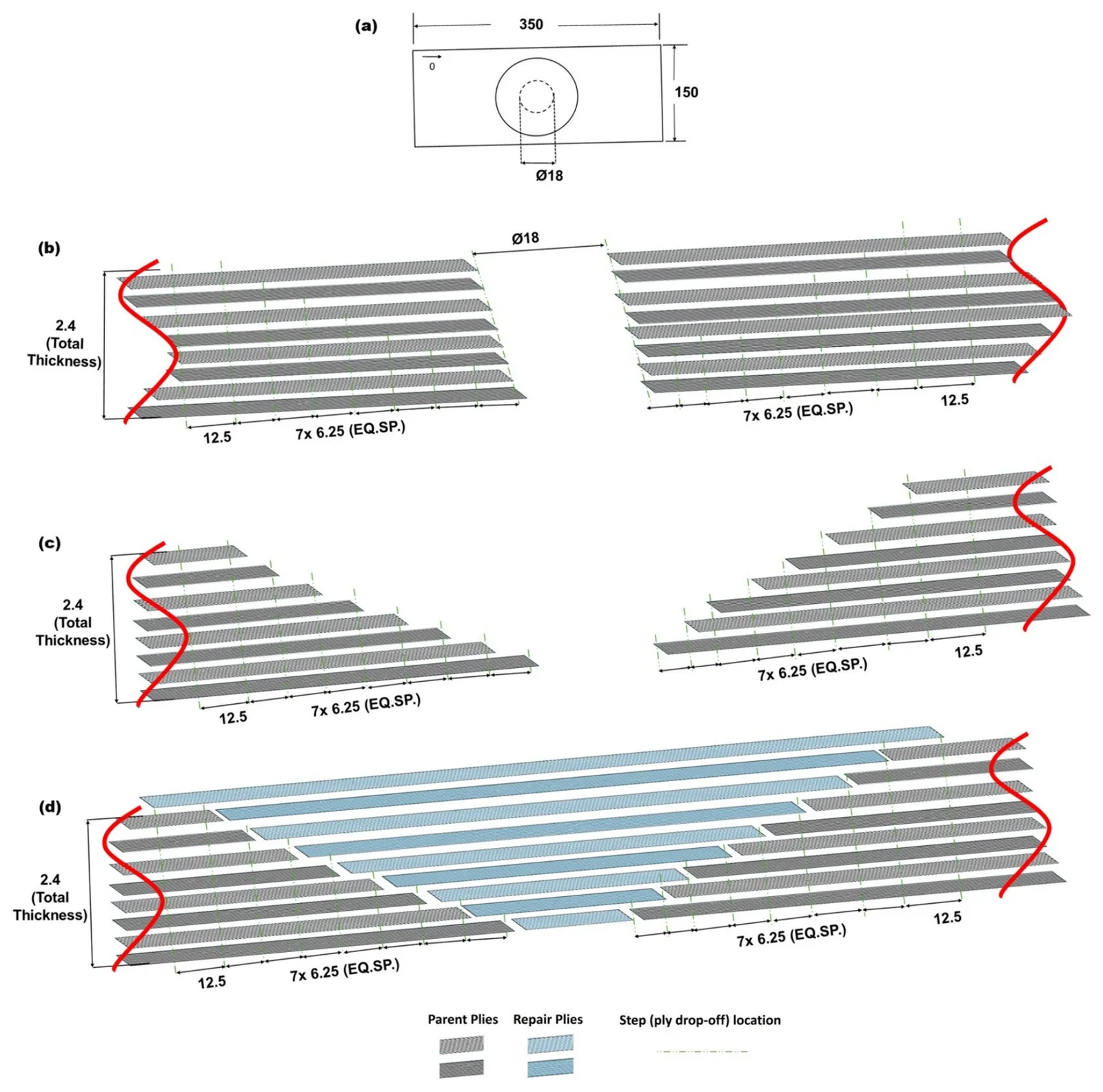
Geometry of the test coupon and the finite element models of the three configurations reproduced from the reference experimental study [12]. (**a**) Nominal coupon geometry, with the Ø18 mm circular cut-out representing the simulated damage and the 0° fibre direction indicated; all dimensions are in mm. (**b**) Damaged configuration, in which the cut-out is left unrepaired. (**c**) Stepped configuration, in which the plies surrounding the cut-out have been machined into a stepped cavity of 6.25 mm per step, giving a stepped length of 43.75 mm on either side. (**d**) Wet lay-up repaired configuration, in which the cavity is filled with repair plies bonded through the adhesive layers shown in light blue, with cohesive surface interactions defined at the interfaces between adjacent plies.

The numerical framework developed in this study is validated against the experimental results reported by Sonat et al. [12] for on-site repaired woven CFRP laminates. All experimental data used in this study (specimen geometry, material properties and measured strengths) are taken from the published results of Sonat et al. [11,12]; no figures or images from these studies are reproduced, and all figures in the present manuscript were prepared by the authors. The reference study investigated stepped repairs manufactured under out-of-autoclave conditions to reproduce practical field-repair constraints. In particular, the wet lay-up repairs were cured at low temperature under vacuum pressure only, conditions that can promote increased porosity and reduced mechanical performance relative to the parent laminate. The experimental study considered both wet lay-up and prepreg step repairs and reported strength-recovery rates of 66% and 76%, respectively [12]. The parent laminate consisted of an eight-ply woven M21/AS4C carbon/epoxy fabric prepreg (Hexcel Corporation, Stamford, CT, USA) with the stacking sequence [±45/0–90/±45/0–90] s and a nominal thickness of 2.4 mm. The panels were machined into specimens measuring 350 mm in length and 150 mm in width, and an 18 mm diameter hole was introduced at the center to represent in-service damage. These dimensions were adopted directly from the reference experimental studies [11,12] so that the numerical predictions could be compared with the measured strengths. The 150 mm width is the largest width that could be loaded uniformly through the grips of the test machine, and it provides a width-to-hole-diameter ratio of 8.3, which limits finite-width effects on the stress field around the cut-out; the 350 mm length provides a sufficient gauge length between the gripped ends to accommodate the stepped repair region (Figure 2). For the stepped configuration, the material surrounding the hole was removed using a three-axis milling machine with a step length of 6.25 mm, corresponding to a nominal scarf angle of 2.8° [12]. Three specimen configurations from the experimental study [12] are reproduced in the present numerical investigation: damaged, stepped, and wet lay-up repaired. These configurations were selected to isolate the effects of damage introduction, material removal, and subsequent repair on the structural response. These three configurations are summarized below, and the corresponding dimensions for each case are presented in Figure 2.

- Damaged: The parent laminate containing the 18 mm central hole without material removal or repair. This configuration represents the baseline damaged condition and characterizes the strength reduction associated with the open-hole geometry.
- Stepped: The laminate after machining the material surrounding the hole into the stepped cavity, but prior to installation of the repair plies. This configuration represents the intermediate state of the repair process and isolates the additional effect of material removal.
- Wet lay-up repaired: The stepped cavity filled with HexForce G0904 D 1070 TCT plain-weave dry carbon fabric (Hexcel Corporation, Stamford, CT, USA) impregnated with HYSOL EA 9396 laminating adhesive (Henkel Corporation, Bay Point, CA, USA) at an adhesive-to-fabric weight ratio of 1:3. Each repair ply was oriented to match the corresponding parent-laminate ply. An additional reinforcement ply, matching the orientation of the outermost repair ply, covered the complete repair area and extended beyond the outermost step.

The experimental tensile tests were conducted using an Instron 5985 testing machine (Instron, Norwood, MA, USA) in accordance with ASTM D3039/D3039M [36] under displacement control at 0.5 mm/min [12]. The loading direction was parallel to the 0° plies. For the damaged, stepped, and wet lay-up repaired specimens, the reported tensile strength was calculated from the maximum load divided by the original cross-sectional area of the parent laminate, 150 × 2.4 mm^2^. The intact-laminate reference strength was obtained separately from standard undamaged coupons and was reported as 600 MPa [12].

The average experimental tensile strengths were 244 MPa for the damaged specimens, 149 MPa for the stepped specimens, and 398 MPa for the wet lay-up repaired specimens, compared with 600 MPa for the intact laminate [12]. These values correspond to strength-recovery ratios of 40.0%, 24.8%, and 66.0%, respectively. A comparison between the finite element model results obtained in the current study and the experimental results from the reference experimental study [12] is presented in Section 3.

### 2.2. Material Properties and Constitutive Models

Three distinct material properties are required by the finite element models: the M21/AS4C parent laminate, the Hexforce G0904 (Hexcel Corporation, Stamford, CT, USA) repair fabric and the HYSOL EA 9396 (Henkel Corporation, Bay Point, CA, USA) adhesive that both impregnates the repair fabric and forms the bond line. Both composite materials are balanced plain weave fabrics and are treated as orthotropic and linear elastic up to damage initiation, with the engineering constants and strengths listed in Table 1. All properties in Table 1 are experimentally determined values; none of them was obtained from manufacturer datasheets or by inverse fitting of the present finite element results. The M21/AS4C properties were measured on the parent laminate in the reference studies [11,12]. The properties of the G0904 fabric impregnated with HYSOL EA 9396 correspond to test results on the cured wet lay-up repair material reported in [11], which are based on the repair-material test program of Ahn and Springer [37]. Because the fabrics are balanced, the in-plane properties are identical in the warp and weft directions. This considerably simplifies the identification of the failure criteria and renders the two in-plane Hashin fibre criteria numerically equivalent. The reduced stiffness and strength of the G0904 repair fabric relative to the M21/AS4C (Hexcel Corporation, Stamford, CT, USA) parent laminate—49.6 GPa against 61.0 GPa in the fibre directions, and 517 MPa against 930 MPa in tension—is a direct consequence of the out-of-autoclave cure and of the higher porosity of the repair zone, reported in the range of 6–8% for wet lay-up repairs against 2–6% for the parent material [12]. This mismatch is retained in the model, since it is the principal source of the stress concentrations that govern the load transfer across the repair.

The adhesive is modelled as a zero-thickness cohesive interface governed by a bilinear traction–separation response rather than as a continuum with an independent bulk constitutive law. This approach follows Sonat et al. [12], who modelled the wet lay-up bond line using a surface-based cohesive interaction rather than discrete cohesive elements, owing to the absence of a well-defined bond-line thickness in adhesive-impregnated fabric repairs. Accordingly, the interfacial stiffnesses cannot be obtained from K_n_ = E/t and K_s_ = K_t_ = G/t, where K_n_, K_s_ and K_t_ denote the normal, first shear, and second shear interfacial stiffnesses, respectively; E and G are the adhesive Young’s and shear moduli, respectively, and tt is the adhesive bond-line thickness. Instead, a penalty stiffness of 10^6^ N/mm^3^ is assigned in all three directions. This value is high enough that the artificial compliance introduced before damage initiation does not affect the global load–displacement response, while remaining low enough to avoid ill-conditioning of the stiffness matrix. The cohesive parameters are summarized in Table 2.

### 2.3. Finite Element Modeling Strategy

All finite element modeling simulations were performed using Abaqus 2022 software (Dassault Systèmes SIMULIA Corp., Johnston, RI, USA). The three configurations shown in Figure 2 were analyzed using a common modelling framework to ensure that differences in the predicted responses arise from the repair geometry and architecture rather than from differences in the numerical formulation.

#### 2.3.1. Meshing of Finite Element Models

The present study uses four-node reduced-integration shell elements (S4R) with a composite section definition. Three Simpson integration points were assigned to each ply, resulting in 24 section points through the thickness of the eight-ply parent laminate. This formulation is consistent with the Hashin damage model implemented in Abaqus, which is formulated for plane-stress elements. It also eliminates the need to discretize each ply through its thickness, substantially reducing the number of degrees of freedom compared with a fully three-dimensional representation. S4R is a general-purpose, shear-deformable shell element: the transverse normal stress σ_33_ is neglected, whereas the transverse shear deformation is retained and the transverse shear stresses are recovered from the section equilibrium. This is the same idealization adopted for laminated skin panels in industrial aircraft stress analysis, where composite skins are routinely represented by shell properties that neglect σ_33_ but retain transverse shear flexibility. It is appropriate for the present problem because the laminate is thin (2.4 mm) relative to its in-plane dimensions, with width-to-thickness ratios of 62.5 for the coupon and 133 for the panel, and because the applied loading acts in the plane of the laminate. Reduced integration was selected because fully integrated thin shell elements are prone to shear locking and because it lowers the computational cost of the fine mesh required in the bonded region; spurious zero-energy (hourglass) modes are suppressed by the hourglass control of the S4R formulation, and their influence is further limited by the 0.1 mm element size in the regions of high stress gradient. Furthermore, plane-stress (two-dimensional) Hashin formulations have been shown to predict the tensile response and the final failure of fibre-reinforced laminates with an accuracy comparable to that of three-dimensional formulations [38].

The stepped geometry in Figure 2c was modelled by locally reducing the number of plies in the composite section by one ply at each step. The shell reference surface was positioned to preserve the physical laminate geometry at each step. The mesh was refined to an element size of 0.1 mm in the stepped and repaired regions and coarsened to 0.3 mm in the undisturbed regions.

**Figure 3:**
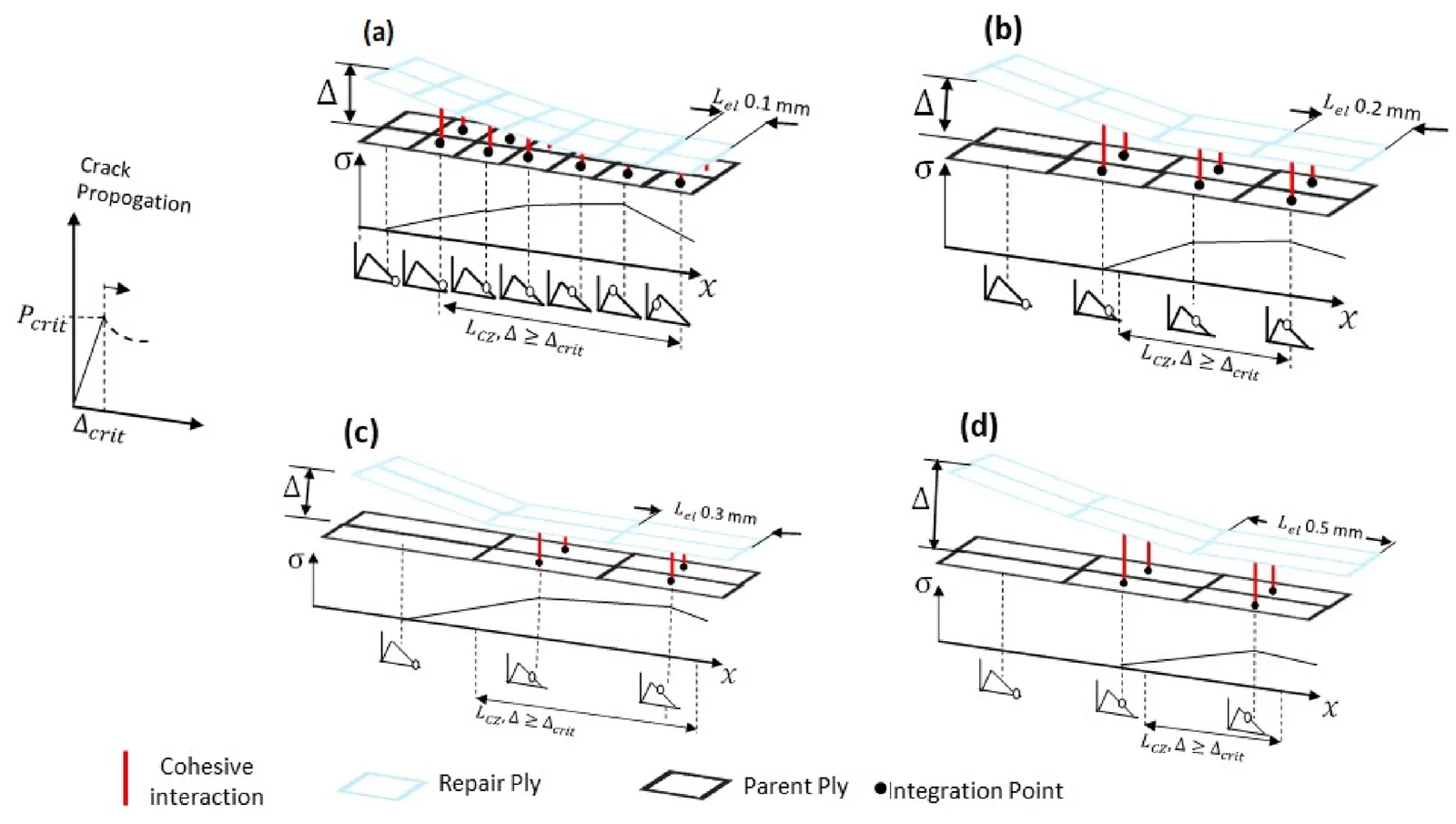
Resolution of the numerical cohesive zone by the in-plane mesh of the bonded region. The numerical cohesive-zone length $L_{CZ}$, over which the separation exceeds the damage-initiation value (∆ ≥ ${∆}_{crit}$) and the traction follows the softening branch of the traction–separation law, is compared with element sizes $L_{el}$ of (**a**) 0.1 mm, (**b**) 0.2 mm, (**c**) 0.3 mm and (**d**) 0.5 mm. Red markers denote the cohesive interactions at the integration points between the repair ply and the parent ply. The predicted cohesive-zone lengths are 0.49 mm in Mode I and 3.59 mm in Mode II; only the 0.1 mm element size satisfies $N_{el}$ ≥ 3 in both modes. Equation numbers in the figure refer to Harper and Hallett [39].

The mesh size in the bonded region was selected based on the cohesive-zone length rather than solely on conventional mesh-convergence considerations. The methodology of Harper and Hallett [39] was used to estimate the characteristic cohesive-zone length and to determine the minimum spatial resolution required within the softening region. In this approach, the analytical cohesive-zone length is expressed as $L_{CZ}$ = M· *E* · *G*_C_/($t^{0}$)^2^, where *E* is the through-thickness modulus of the adherend, *G*_C_ and $t^{0}$ are the critical fracture energy and the interface strength of the corresponding mode (Table 2), and M is a model-dependent parameter of order unity [39,40]. The length of the numerical cohesive zone is taken as half of the smallest analytical estimate, $L_{CZ}$ = 0.5·min($L_{CZ}$) [39]. Using the through-thickness modulus of the repair fabric (Table 1), which gives the shorter and therefore more restrictive estimate, the numerical cohesive-zone lengths are approximately 0.49 mm in Mode I and 3.59 mm in Mode II. Their results indicate that approximately two to three elements within the numerical cohesive zone are sufficient to accurately reproduce the load–displacement response [11,12]. Based on the minimum cohesive-zone length predicted for the present material and interface properties, an element size of 0.1 mm was adopted in the stepped and repaired regions. This ensured that at least three elements were present within the cohesive zone under the investigated loading conditions. With this element size, 4.9 and 35.9 elements lie within the Mode I and Mode II cohesive zones, respectively, compared with the requirement of $N_{el}$ ≥ 3. As illustrated in Figure 3, coarser element sizes of 0.2, 0.3 and 0.5 mm would place only 2.5, 1.6 and 1.0 elements within the Mode I cohesive zone, which is insufficient to resolve the softening region of the traction–separation law.

A mesh-sensitivity study was subsequently performed by varying the element size in the bonded region from 0.5 to 0.05 mm. The predicted peak load varied by less than 2.5% for element sizes below 0.1 mm, confirming that further mesh refinement had a negligible effect on the global response. For the wet lay-up repaired configuration, the predicted peak loads obtained using element sizes of 0.5, 0.3, 0.2, 0.1, and 0.05 mm were 152.8, 145.0, 139.6, 134.6, and 133.4 kN, respectively. Relative to the finest-mesh result, the corresponding deviations were 14.5%, 8.7%, 4.6%, and 0.9%, while the 0.05 mm mesh served as the reference for comparison. The difference between the results obtained with the 0.1 and 0.05 mm meshes was approximately 0.9%, indicating that further mesh refinement had a limited effect on the predicted peak load. Accordingly, 0.1 mm was selected as the final element size to provide an appropriate balance between numerical accuracy and computational efficiency.

#### 2.3.2. Interface Modelling

For the wet lay-up repair configuration in Figure 2d, the interfaces between the repair patch and the corresponding parent-laminate steps were modelled using surface-based cohesive interactions. This approach is consistent with the reference experimental configuration [12], in which the adhesive-impregnated repair fabric does not form a distinct bondline with a well-defined thickness. The cohesive interaction was defined using the damage initiation and evolution criteria. Following damage-induced separation, tangential contact was governed using a penalty formulation with a friction coefficient of 0.3. Cohesive behavior was assigned only to the interfaces between the repair patch and the parent laminate. No debonding was introduced between the additional reinforcement ply and the outermost repair ply because experimental observations indicated no interfacial failure between these layers. The configurations in Figure 2b,c do not contain bonded interfaces; therefore, their failure response is governed solely by the intralaminar damage criteria.

#### 2.3.3. Loading and Boundary Conditions

The complete 350 × 150 mm specimen was modelled without symmetry reduction. Although the overall laminate and repair geometry are nominally symmetric in plan view, the stepped configuration is asymmetric through the thickness. A full-model representation was therefore adopted to capture the resulting stress and damage distributions without imposing artificial symmetry constraints. This also enables the asymmetry of the damage field to be examined directly. One end of the specimen was fully constrained over the tab region to reproduce the fixed-grip condition. At the opposite end, an axial displacement of 4.0 mm was prescribed through a reference point kinematically coupled to the end edge, thereby reproducing the displacement-controlled ASTM D3039 test configuration. The loading direction was aligned with the 0° laminate direction.

The analyses were conducted using the Newton–Raphson solution scheme with automatic incrementation. Viscous regularization was introduced to improve numerical stability during the softening response associated with intralaminar and interfacial damage. A viscosity coefficient of ${1\times 10}^{-5}$ was assigned to both the cohesive interaction and the composite damage evolution. The influence of numerical stabilization was monitored through the viscous dissipation energy (ALLCD). Throughout the analysis, ALLCD remained below 2% of the total internal energy (ALLIE) up to final failure, indicating that the stabilization had a limited contribution to the overall energy balance. The selected regularization parameter therefore provided numerical stability while maintaining the predicted structural response.

### 2.4. Damage Initiation and Evolution Criteria

Two distinct damage mechanisms are considered in the repaired laminates: intralaminar damage within the composite plies and interfacial damage at the repair–parent laminate interface. The two mechanisms are modelled independently using the constitutive formulations described in the following subsections.

#### 2.4.1. Intralaminar Damage

Intralaminar damage initiation is evaluated using stress-based failure criteria defined in the material coordinate system of each ply. For the reference woven-fabric repair model, the failure formulation distinguishes four fibre-dominated failure modes associated with the warp and weft directions [11,12]. The corresponding failure indices are expressed as the following.

Warp-fibre tension $\left(\sigma_{11}\geq 0\right)$:

(1) $$FI_{Warp}^{t}={\left(\frac{\sigma_{11}}{X_{T}}\right)}^{2}+{\left(\frac{\tau_{12}}{S_{12}}\right)}^{2}$$

Warp-fibre compression $\left(\sigma_{11}<0\right)$:

(2) $$FI_{Warp}^{c}={\left(\frac{\sigma_{11}}{X_{C}}\right)}^{2}$$

Weft-fibre tension $\left(\sigma_{22}\geq 0\right)$:

(3) $$FI_{\mathrm{Weft}\;}^{t}={\left(\frac{\sigma_{22}}{Y_{T}}\right)}^{2}+{\left(\frac{\tau_{12}}{S_{12}}\right)}^{2}$$

Weft-fibre compression $\left(\sigma_{22}<0\right)$:

(4) $$FI_{Weft}^{c}={\left(\frac{\sigma_{22}}{Y_{C}}\right)}^{2}$$

Here, $X_{T}$ and $X_{C}$ denote the tensile and compressive strengths in the 1-direction, $Y_{T}$ and $Y_{C}$ are the corresponding strengths in the 2-direction, and $S_{12}$ is the in-plane shear strength. Damage initiation is assumed when the governing failure index reaches unity, with the overall failure index at a material point defined as the maximum of the four mode-specific indices.

In contrast to this fibre-dominated formulation, the present model employs the Hashin damage formulation available in Abaqus/Standard. Accordingly, both fibre- and matrix-dominated failure mechanisms are considered, including fibre tension, fibre compression, matrix tension, and matrix compression. For a plane-stress state, the corresponding Hashin initiation criteria are written as follows:

Fibre tension $\left(\sigma_{11}\geq 0\right)$:

(5) $$F_{f}^{\pm }={\left(\frac{\sigma_{11}}{X_{T}}\right)}^{2}+\alpha {\left(\frac{\tau_{12}}{S_{12}}\right)}^{2}$$

Fibre compression $\left(\sigma_{11}<0\right)$:

(6) $$F_{f}^{c}={\left(\frac{\sigma_{11}}{X_{C}}\right)}^{2}$$

Matrix tension $\left(\sigma_{22}\geq 0\right)$:

(7) $$F_{m}^{t}={\left(\frac{\sigma_{22}}{Y_{T}}\right)}^{2}+{\left(\frac{\tau_{12}}{S_{12}}\right)}^{2}$$

Matrix compression $\left(\sigma_{22}<0\right)$:

(8) $$F_{m}^{c}={\left(\frac{\sigma_{22}}{2S_{23}}\right)}^{2}+\left[{\left(\frac{Y_{C}}{2S_{23}}\right)}^{2}-1\right]\frac{\sigma_{22}}{Y_{C}}+{\left(\frac{\tau_{12}}{S_{12}}\right)}^{2}$$

Damage is initiated in a particular mode when its corresponding failure index reaches unity. The parameter α controls the contribution of the in-plane shear stress to fibre tensile failure and is set to unity in the present analysis.

The adoption of the four-mode Hashin formulation is consistent with the S4R shell formulation used in the present study and enables fibre- and matrix-dominated damage to be evaluated within the plane-stress assumption. This is particularly relevant to the stepped repair configuration, where the local reduction in laminate thickness introduces non-uniform stress distributions in the vicinity of the ply-drop regions. Retaining the matrix failure modes therefore provides a more complete representation of the local damage state than a fibre-dominated criterion alone.

Following damage initiation, stiffness degradation is governed by a progressive energy-based evolution law rather than by instantaneous material-property degradation. Separate damage variables are introduced for the fibre and matrix failure modes, and the corresponding constitutive stiffnesses are progressively reduced as the equivalent displacement evolves from its value at damage initiation to that at complete failure. The evolution parameters are defined from the mode-dependent fracture energies listed in Table 1. The characteristic element length is incorporated into the damage evolution formulation to regularize the softening response and to provide an energy-consistent representation of damage progression. The resulting degradation follows the progressive damage formulation implemented in SIMULIA Abaqus/Standard.

#### 2.4.2. Interfacial Damage

Interfacial failure between the repair patch and the parent laminate is modelled using a surface-based cohesive interaction with a bilinear traction–separation response. Prior to damage initiation, the interface is assumed to behave elastically, with the normal and two tangential traction components related to the corresponding separations according to

(9) $$t_{i}=K_{i}\delta_{i},i=n,s,t$$

where $t_{i}$ and $\delta_{i}$ denote the traction and relative displacement components in the normal and two tangential directions, respectively, and $K_{i}$ represents the corresponding penalty stiffness.

Damage initiation is governed by the quadratic nominal-stress criterion

(10) $${\left(\frac{\left\langle t_{n}\right\rangle }{t_{n}^{0}}\right)}^{2}+{\left(\frac{t_{s}}{t_{s}^{0}}\right)}^{2}+{\left(\frac{t_{t}}{t_{t}^{0}}\right)}^{2}=1,$$

where $t_{n}^{0},\;t_{s}^{0}$, and $t_{t}^{0}$ are the nominal interface strengths. The Macaulay operator ⟨⋅⟩ excludes compressive normal traction from contributing to damage initiation.

The post-initiation response is governed by a mixed-mode fracture-energy formulation based on the Benzeggagh–Kenane (B-K) criterion as

(11) $$G_{c}=G_{Ic}+\left(G_{IIc}-G_{Ic}\right){\left(\frac{G_{II}+G_{III}}{G_{I}+G_{II}+G_{III}}\right)}^{\eta },$$

where $G_{I},\;G_{II}$, and $G_{III}$ denote the mode-I, mode-II, and mode-III components of the dissipated fracture energy, respectively, $G_{Ic}$ and $G_{IIc}$ are the corresponding critical fracture energies, and η is the B-K interaction parameter. The material parameters adopted for the cohesive interface are listed in Table 2. The value of η is selected based on the available experimental or literature data for the adhesive system [41].

The cohesive parameters were selected as follows. The interface strengths ($t_{n}^{0}$ = 55 MPa, $t_{s}^{0}$ = $t_{t}^{0}$ = 26 MPa) and the mode I and mode II fracture energies ($G_{Ic}$ = 0.4 N/mm, G_*IIc*_ = 0.7 N/mm) in Table 2 are those of the HYSOL EA 9396 wet lay-up bond line characterized in the reference studies [11,12], and the mode III fracture energy was taken equal to the mode II value ($G_{IIIc}$ = $G_{IIc}$), as is common when no separate mode III data are available. The B–K interaction parameter was set to η = 1.5 [41]. The penalty stiffness of 10^6^ N/mm^3^ is a numerical parameter rather than a material property; it was chosen high enough for the initial compliance of the interface to be negligible compared with that of the adjacent plies, and within this range the peak load of cohesive models is known to be insensitive to its exact value [39,40]. The sensitivity of the predicted residual strength to these parameters can be assessed from the loading of the interface. Because the panels are loaded in in-plane tension, the repair–parent interfaces are loaded predominantly in shear (modes II and III). For mode mixities close to pure shear, Equation (11) gives $G_{c}$ ≈ $G_{IIc}$ irrespective of η, so that the predicted strength is expected to be only weakly sensitive to η and to $G_{Ic}$, but directly sensitive to the shear strength $t_{s}^{0}$ and to $G_{IIc}$. Since all configurations share the same interface, a change in these two parameters would shift the failure loads of all configurations in the same direction; it would therefore affect the absolute values of strength retention factor more than the ranking of the configurations, which is the basis of the present conclusions (see also Section 3.3.2).

**Figure 4:**
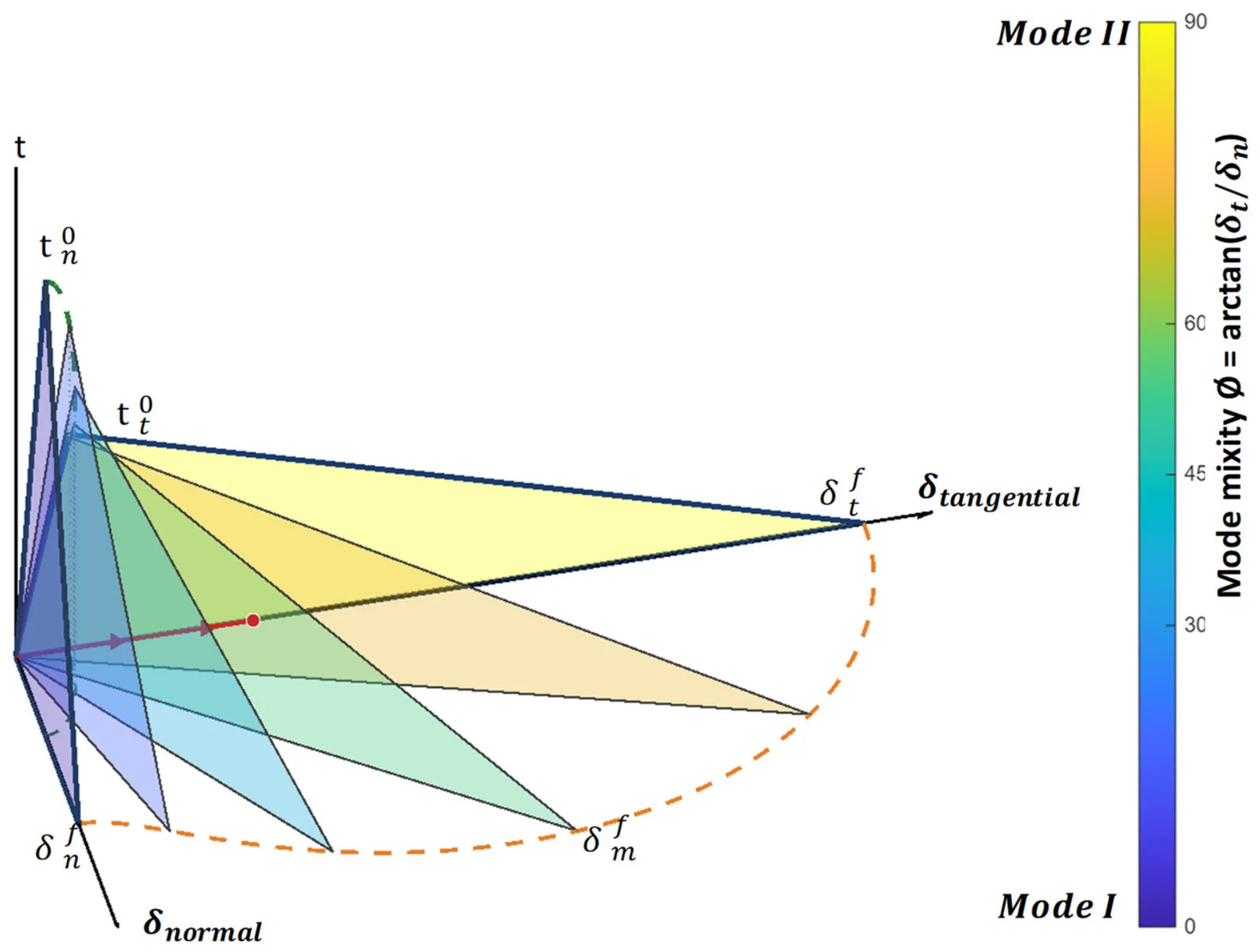
Mixed-mode bilinear traction–separation surface adopted for the adhesive bond line, constructed from the cohesive properties of Table 2. Each bilinear response corresponds to a fixed mode mixity angle φ = arctan(δ_t_/δ_n_), indicated by its color; the two emphasized outlines are the pure Mode I and pure Mode II responses. The dashed green curve (on t-$\delta_{normal}$ plane) is the locus of the peak tractions, that is, the damage initiation envelope, and the dashed orange curve on the base plane is the locus of the separations at complete decohesion. The red line shows the secant unloading and reloading path followed by a partially damaged interface. The ratio of the onset separation to the failure separation is exaggerated for clarity; with the penalty stiffness of 10^6^ N/mm^3^, the actual ratio is of the order of 10^−3^.

**Figure 5:**
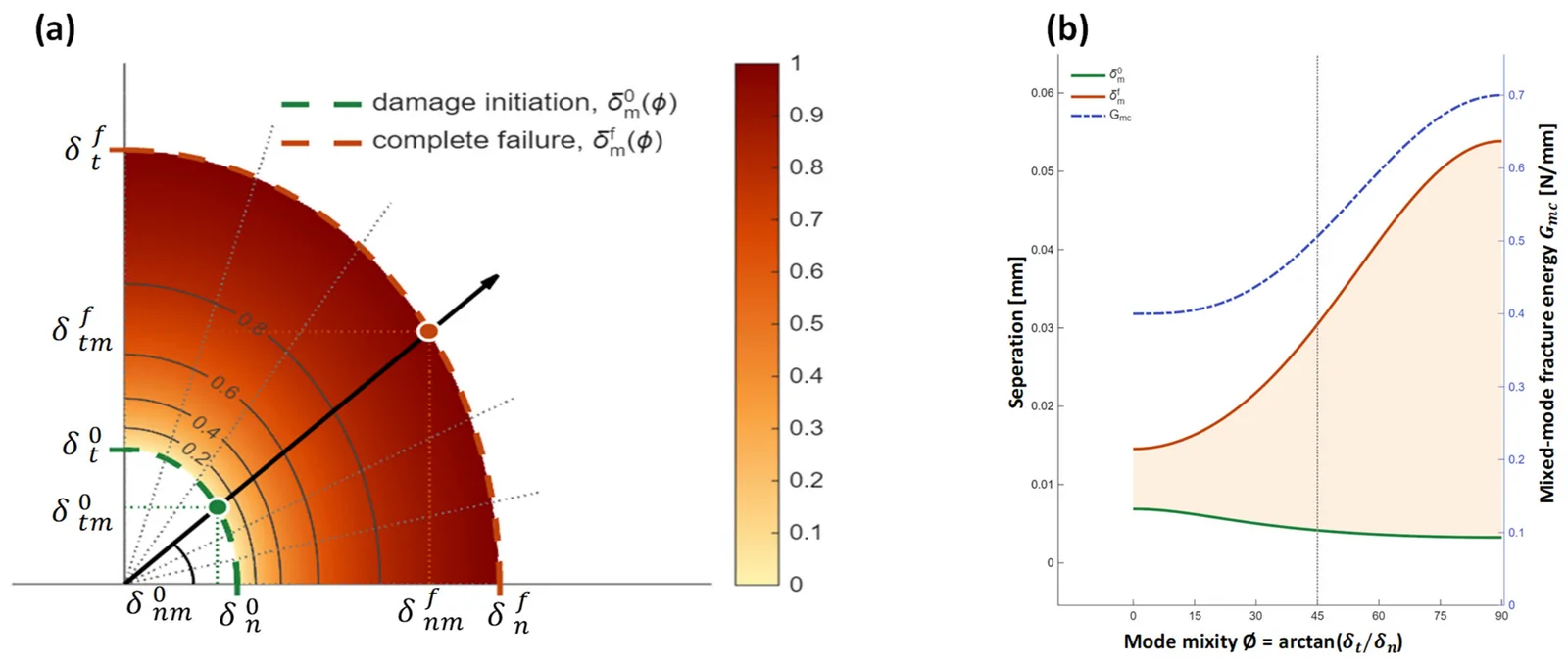
Mixed-mode damage description of the adhesive bond line. (**a**) Scalar damage variable D in the separation plane, bounded by the quadratic initiation envelope (dashed green) and the energetic failure envelope (dashed orange); the thin contours are lines of constant damage and the arrow indicates a proportional loading path at constant mode mixity, whose intersections with the two envelopes define the component separations δ^0^_nm_, δ^0^_tm_ and δᶠ_nm_, δᶠ_tm_. (**b**) Dependence of the equivalent separations δ^0^_m_, δᶠ_m_, and of the mixed-mode fracture energy $G_{mc}$ on the mode mixity angle.

For the bilinear traction–separation response, the effective separation at complete decohesion is determined from the prescribed mixed-mode fracture energy. The scalar damage variable is subsequently defined as

(12) $$D=\frac{\delta_{m}^{f}\left(\delta_{m}-\delta_{m}^{0}\right)}{\delta_{m}\left(\delta_{m}^{f}-\delta_{m}^{0}\right)},$$

where $\delta_{m}$ is the current effective separation, while $\delta_{m}^{0}$ and $\delta_{m}^{f}$ denote the effective separations at damage initiation and complete decohesion, respectively. The damage variable evolves from D=0 at damage initiation to D=1 at complete separation. The degradation is irreversible, such that the maximum attained damage state is retained during subsequent unloading and reloading. The mixed-mode bilinear traction–separation law adopted for the adhesive bond line is illustrated in Figure 4, while the corresponding evolution of the scalar damage variable and its dependence on the mode mixity angle are presented in Figure 5.

## 3. Results and Discussion

### 3.1. Validation of the Numerical Model

The numerical framework described in Section 2 is validated against the three configurations of the reference experimental study [12] for which the present modelling strategy is applicable: the damaged, the stepped and the wet lay-up repaired laminate. Together these three cases exercise the two constitutive models independently. The damaged and stepped configurations contain no bond line, so their predicted response depends solely on the intralaminar Hashin criteria and on the shell representation of the stepped geometry; the wet lay-up repaired configuration adds the cohesive interface and therefore tests the mixed-mode formulation. Agreement across all three cases consequently demonstrates that the framework is not calibrated to reproduce a single result.

**Figure 6:**
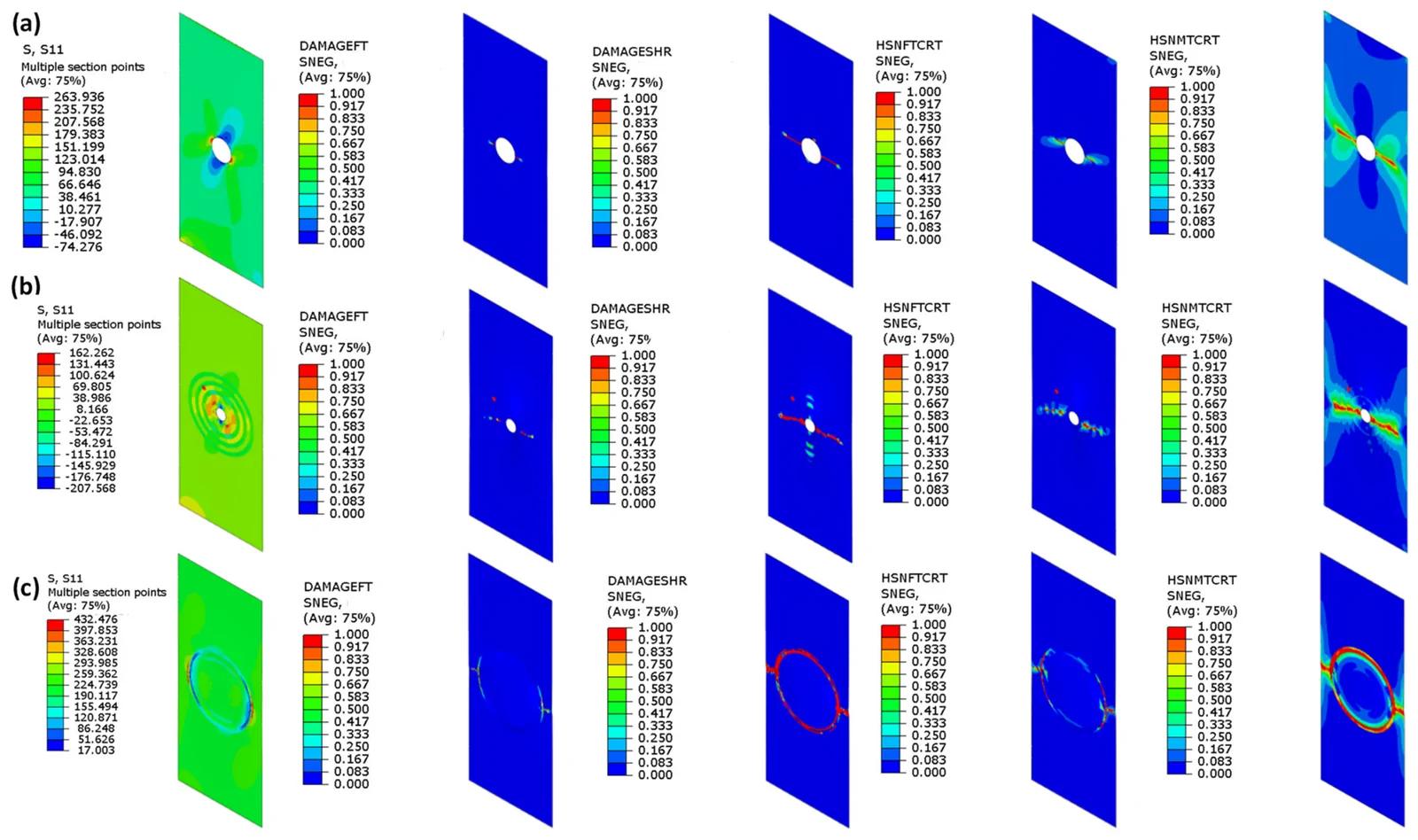
Predicted stress and damage state at the onset of final failure for the (**a**) damaged, (**b**) stepped and (**c**) wet lay-up repaired configurations. For each configuration, from left to right: axial stress σ_11_, fibre tensile damage variable (DAMAGEFT), shear damage variable (DAMAGESHR), Hashin fibre tensile initiation index (HSNFTCRT) and Hashin matrix tensile initiation index (HSNMTCRT). All quantities are evaluated on the bottom surface of the shell section (SNEG, fraction = −1.0). Initiation indices are capped at unity, and a value of unity in the damage variables denotes complete degradation of the corresponding stiffness component. The stress values are local and are not to be compared directly with the net-section strengths of Table 3.

The predicted strength of each configuration is obtained from the maximum reaction force at the loaded end, divided by the initial cross-sectional area of 150 mm × 2.4 mm, so that it is defined identically to the experimental strength reported in the reference study [12]. This distinction is necessary because the peak values of the axial stress field which are presented in Figure 6 are local quantities at the edge of the cut-out and exceed the net-section strength by approximately the stress concentration factor of the laminate. Figure 6 also provides the damage states for the three configurations. For the damaged configuration the ratio of the local peak of 264 MPa to the predicted net-section strength of 228.3 MPa is 1.15, of the same order as the concentration factor of 1.25 adopted analytically for this quasi-isotropic woven laminate in the reference study. The contour values of Figure 6 are therefore used only as an indication of the stress distribution. Table 3 summarizes the comparison of the predicted tensile strengths with the experimental results of [12].

According to the results, the numerical finite element model reproduces the experimental findings [12] of the three configurations and predicts each strength within 6.4% accuracy. This represents an improvement over the 10% accuracy reported for the three-dimensional solid model of the reference study, while employing a shell discretization at a substantially lower computational cost. Two aspects of the agreement are notable. First, the deviation is almost identical for the three configurations, at −6.4%, −6.4% and −6.1% respectively. This offset is consistent across cases with different failure mechanisms, suggesting a uniform bias rather than a model-specific error. If either model were inaccurate alone, the damaged and stepped configurations would deviate differently from the repaired one. Because the same offset is present in the damaged and stepped configurations, which contain no cohesive interface, the bias cannot originate from the interfacial model and must be attributed to features common to all three models. Three such sources are identified. First, the intralaminar properties in Table 1 are mean values from separate material characterization [11,12], whereas the fibre-dominated strength of the batch used for the repair tests was not reported; since final failure in all three configurations is fibre-dominated in the parent laminate, any difference between these strengths scales the three predicted failure loads by a similar fraction. Second, the fibre-tension criterion of Equation (5) is used with α = 1, so that the full in-plane shear contribution is added to the fibre failure index; in the ±45° plies, where $\tau_{12}$ is significant, this advances damage initiation and gives a systematically conservative strength prediction. Third, the shell idealization neglects the through-thickness constraint and uses the nominal laminate thickness of 2.4 mm, and the grip region is idealized as fully fixed; these simplifications act in the same way in all three models. The influence of viscous regularization is negligible in comparison, as the stabilization energy remained below 2% of the internal energy (Section 2.3.3). A uniform underprediction of about 6% is conservative from a design perspective, and because it is common to all configurations, it does not affect the strength ratios on which the comparisons in this study are based. Second, the predicted strength ratios reproduce the measured ones almost exactly: the ratio of the stepped to the damaged strength is 0.611 both experimentally and numerically, and the ratio of the wet lay-up repaired to the damaged strength is 1.638 against the measured value of 1.631. The relative performance difference of the three configurations, which matters most when assessing a repair procedure, is captured to be lower than 0.5%. The close agreement between the finite element model of the present study and the previous experimental findings [12] validates the finite element modeling strategy.

### 3.2. Failure and Damage Characteristics

The axial stress field in Figure 6a shows the expected concentration around the open hole, with a maximum tensile stress of 264 MPa at the transverse extremities of the hole and a compressive stress of −74 MPa at the longitudinal poles. Fibre tensile damage remains localized near the transverse hole boundary, indicating a fibre-dominated failure across the net section. Matrix damage extends over a wider region but does not govern the predicted failure, consistent with the experimentally observed fracture perpendicular to the loading direction [12].

The stepped configuration in Figure 6b exhibits a markedly different stress distribution, with tensile and compressive extremes of 162 and −208 MPa, respectively. The dominant compressive response reflects the bending–extension coupling induced by the asymmetric stepped geometry and reproduced through the shell-section offset. Damage localizes primarily along the step edges, and the predicted strength is the lowest among the three configurations, consistent with the previous experimental results [12].

For the wet lay-up repaired configuration in Figure 6c, damage is concentrated around the perimeter of the bonded region. Interfacial shear damage develops preferentially along the outer repair boundary, whereas fibre tensile damage remains comparatively limited. This indicates that failure is initially governed by interfacial degradation before propagating into the parent laminate, consistent with the experimental failure sequence reported by Sonat et al. [12]. The predicted localization of damage along the bonded region also agrees with the experimentally observed dependence of failure on the local fibre orientation. The three configurations provide complementary validation of the intralaminar and interfacial damage formulations and support the application of the proposed framework to the panel-level parametric study.

Two limitations should nevertheless be noted. First, the experimentally observed porosity of the wet lay-up repair is not explicitly modelled; its influence is incorporated through the effective mechanical properties assigned to the repair material. Consequently, specimen-to-specimen variations associated with porosity are not resolved. Second, the conventional shell formulation does not resolve through-thickness stresses and therefore does not account for interlaminar failure. This limitation is not expected to alter the comparative conclusions of the parametric study, for three reasons. (i) The dominant interlaminar failure mechanism of a bonded repair, debonding of the repair–parent interface, is represented explicitly by the cohesive interaction, which includes both the normal (peel) and the shear traction components (Equations (9)–(11)); only delamination between the plies of the parent laminate and of the patch is not resolved. (ii) The through-thickness stresses that drive such delamination are confined to boundary layers at the ply terminations, whose extent is of the order of the laminate thickness (≈2.4 mm) and therefore considerably smaller than the narrowest ligament investigated (18 mm); the ligament itself is loaded predominantly in-plane, a membrane state that the shell formulation captures. (iii) The same formulation is applied to all configurations, so any omitted interlaminar contribution affects them in a similar manner and the ranking of the configurations is preserved. For the most closely spaced transverse repairs, where the boundary layers of the two repairs are nearest to each other, delamination within the parent laminate could slightly advance final failure, and the residual strengths of these configurations may be marginally overestimated. This limitation is acceptable for the present in-plane tensile loading, but the framework should be applied with caution to loading conditions involving significant out-of-plane stresses.

The extent to which this validation supports the panel-level study should also be stated explicitly. The dual-repair panels are built from the same materials, interface definition, element formulation and mesh density, so that the panel model differs from the validated one only in its geometry and in the interaction between the stress fields of adjacent repairs. The validation does not cover the coalescence of the interfacial damage fields of two repairs, the load redistribution through the ligament and the interaction between the repairs and the free edges have not been verified experimentally, since, to the authors’ knowledge, no test data for dual stepped-lap repairs are available in the literature. The panel-level results should therefore be regarded as predictions of a validated modelling approach rather than as experimentally validated results. Comparisons between configurations, which rely on relative strengths, are considered more reliable than the absolute values of strength retention factor. Experimental testing of selected dual-repair configurations, such as the closest and the most favorable transverse spacings, is identified as the key next step.

### 3.3. Panel-Level Parametric Study: Multi-Site Repair Scenarios

The validated numerical framework is subsequently extended to a panel-level parametric investigation of multi-site stepped-lap repairs. The study examines the effects of repair-site spacing, patch stacking sequence, and relative spatial arrangement on load transfer, stress redistribution, and damage interaction. A 400 × 320 mm composite panel is modelled under uniaxial tensile loading. The transverse edges of the panel are left free, allowing unconstrained transverse deformation under tensile loading. The loading direction is parallel to the global xx-axis and coincides with the principal tensile loading direction. This boundary-condition definition represents a displacement-controlled tensile loading configuration while avoiding artificial transverse restraint of the panel.

The free-edge condition was selected deliberately as a lower bound on the support provided by the surrounding structure. In aircraft structures, skin panels are attached to spars, ribs, frames or stringers through single or double rows of fasteners, or are bonded directly to stiffening elements, so that their edges behave between the idealized simply supported and clamped conditions. In panel-level stress analysis, both idealized conditions are commonly analyzed and the design value is taken as their average, or as a weighted combination calibrated against test results. A free edge, in contrast, provides neither transverse nor rotational restraint and represents the weakest possible support: load that cannot be redistributed into adjacent structure must be carried by the panel itself, which yields the lowest residual strength for a given repair configuration. This choice is also motivated by the multi-site repair scenario considered here. When the neighboring panels, or the adjacent regions of the same panel, are themselves repaired, their response may deviate from the design assumption, and the support they provide can no longer be assumed to be simply supported or clamped; in some scenarios even a simple support cannot be guaranteed, and the boundary conditions cannot be defined reliably. Adopting the free-edge condition avoids relying on a support stiffness that may not be present. The results reported below should therefore be interpreted as conservative estimates relative to panels with supported edges; in particular, the repair-to-free-edge interaction identified at large spacings would be expected to weaken when the edges are restrained.

Uniaxial tension was selected because it is the loading case in which the net-section load path through the ligament between two repairs is most directly challenged, and because it is the loading case for which the modelling approach was validated. Under in-plane shear or compression, which are also relevant for fuselage and wing skins, the failure mechanisms would differ as these loads may trigger local or global buckling and introduce peel stresses at the repair edges, which the present conventional shell model with free edges is not intended to capture. The assumptions also influence the predicted load redistribution and stress concentrations. Because the longitudinal edges are free, the load diverted around the repairs must be carried entirely within the panel width, so the stress concentrations in the ligament and in the strips between the repairs and the free edges are higher than in a panel whose edges are supported by adjacent structure. This is expected to amplify mainly the repair-to-free-edge interaction at large spacings. The repair-to-repair interaction at small spacings is governed by the ligament and is therefore less affected. The predicted failure mechanisms, interfacial decohesion at the repair perimeter followed by fibre-dominated failure of the parent laminate, are consistent with those observed for the validated single-repair coupons [12], but their sequence under combined loading remains to be established.

**Figure 7:**
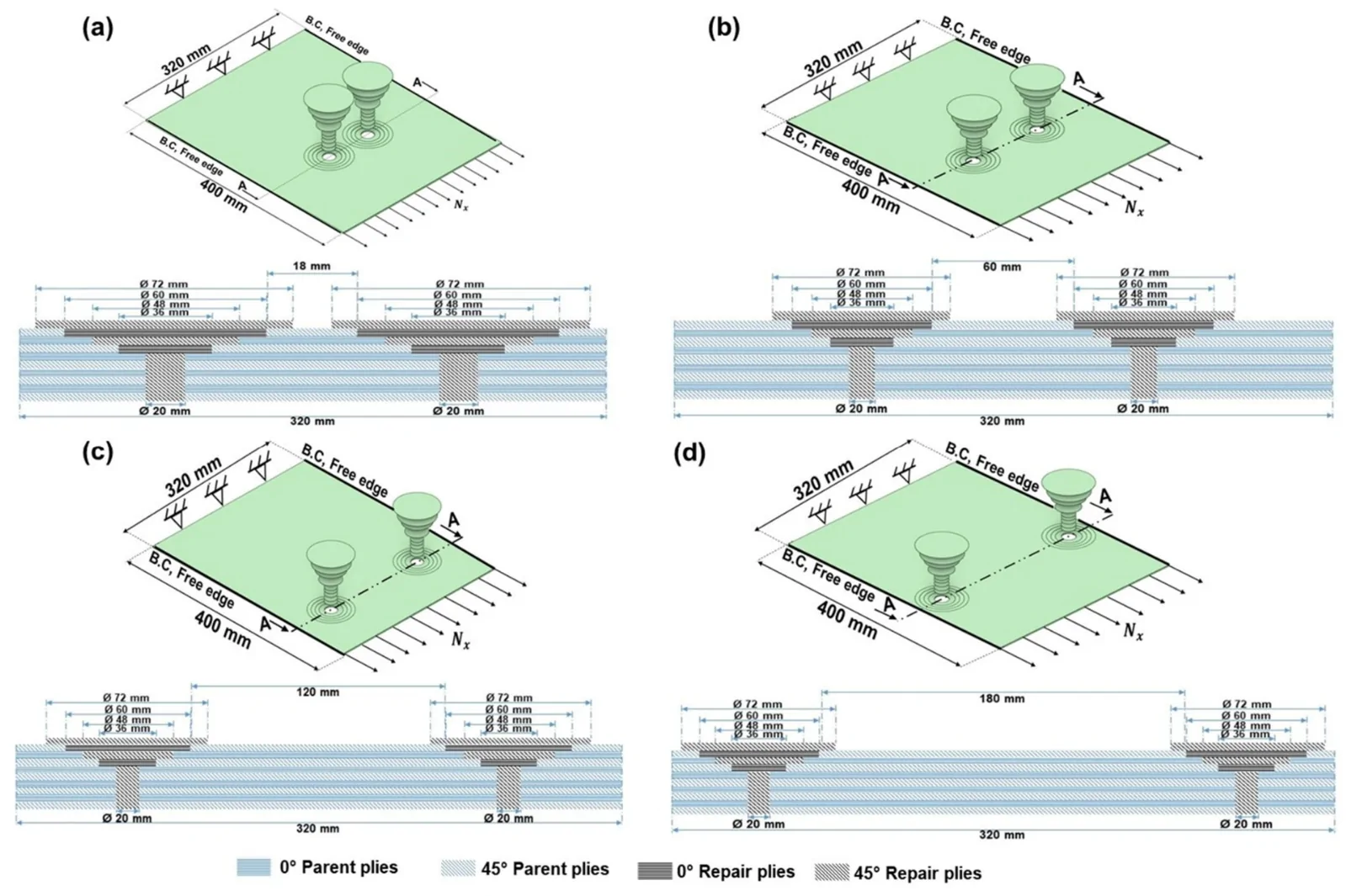
Geometrical configurations and boundary conditions for the transversely arranged (side-by-side) multi-site repair scenarios (Scenarios 1–12). The ligament distance L is measured edge-to-edge between the outer boundaries of the stepped cavities (Ø60 mm) along section A–A. The schematics illustrate four distinct spacing intervals: (**a**) L = 18 mm (interacting proximity; 6 mm gap between the outermost repair plies), (**b**) 60 mm, (**c**) 120 mm, and (**d**) 180 mm isolated ligament spacing.

The parametric study comprises 15 distinct repair scenarios. The first 12 scenarios consider two repaired sites placed side by side across the panel width, i.e., transverse to the loading direction, as illustrated in Figure 7. These configurations are designed to investigate the interaction between neighboring repairs that share the same loaded cross-section. Four ligament distances, L, are considered as 18, 60, 120, and 180 mm. The ligament distance L is defined as the edge-to-edge distance between the outer boundaries of the two stepped cavities (Ø60 mm), measured along the line joining the repair centers (section A–A in Figure 7); it therefore corresponds to the width of un-machined parent laminate retained between the two repairs. Because the outermost repair ply (Ø72 mm) extends 6 mm beyond the cavity boundary, the clear gap between the outermost repair plies is L—12 mm. For the closest configuration (L = 18 mm), the two repair regions are thus separated by an 18 mm un-repaired ligament, while the outermost repair plies are 6 mm apart. The larger values of L represent progressively greater separation between the repaired regions and therefore provide a basis for assessing the evolution of repair–repair interaction with increasing spacing.

**Figure 8:**
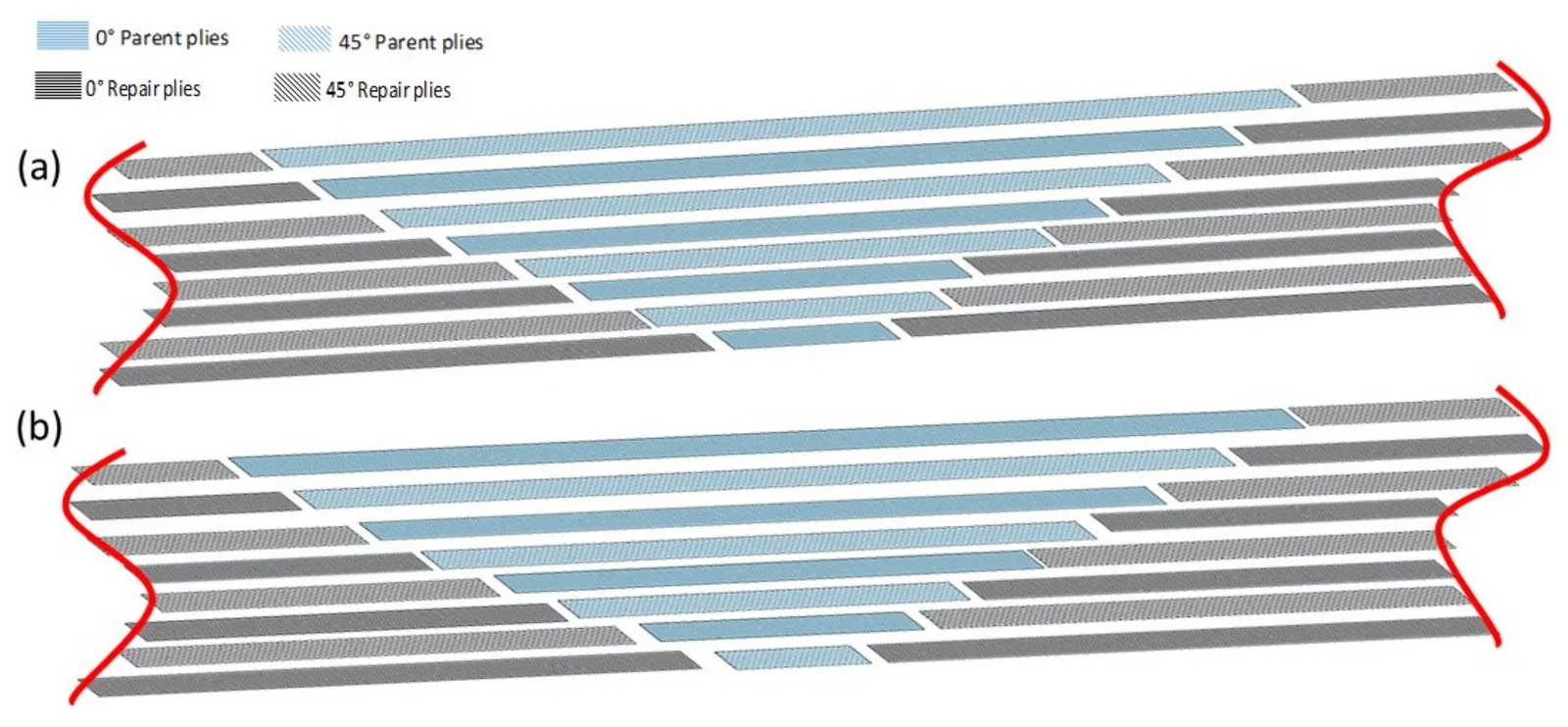
Schematic representation of the patch stacking sequences evaluated in the parametric study, illustrating the through-thickness architecture of (**a**) the matched lay-up configuration and (**b**) the baseline overlapping lay-up.

For each transverse arrangement, three different repair patch stacking sequences are investigated. Scenarios 1–4 employ the baseline patch lay-up, Scenarios 5–8 employ a stiffness-matched lay-up, and Scenarios 9–12 employ the more compliant [±45°/±45°] angle-ply lay-up. Thus, the first 12 cases constitute a systematic 3 × 4 parameter matrix, combining three patch architectures with four transverse ligament distances. The differences in ply arrangement and stiffness distribution between the baseline and stiffness-matched repair architectures are illustrated in Figure 8; the compliant patch follows the same step geometry with all repair plies oriented at ±45°.

**Figure 9:**
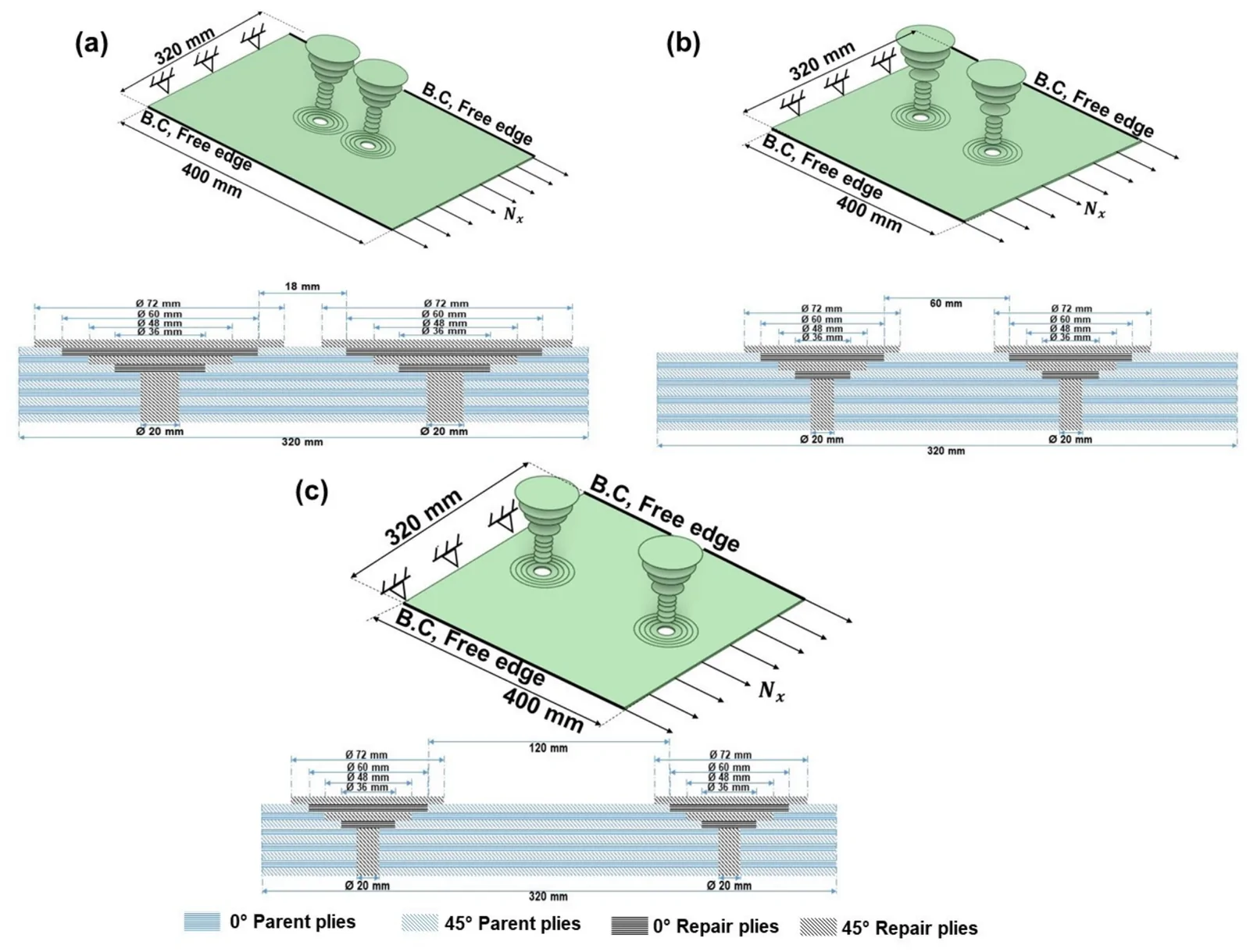
In-line multi-site repair configurations (Scenarios 13–15) subjected to longitudinal tension. The two repairs are aligned with the loading direction, with ligament spacings of (**a**) 18 mm, (**b**) 60 mm, and (**c**) 120 mm.

The remaining three scenarios, 13–15, investigate an in-line repair arrangement, in which the two repaired sites are positioned along the loading direction, as shown in Figure 9. Ligament distances of 18, 60, and 120 mm are considered for this configuration, while the baseline patch lay-up is retained. These cases provide a direct comparison between transverse and in-line repair-site arrangements and enable the influence of the remaining un-repaired ligament on load transfer and stress redistribution to be assessed.

Accordingly, the 15 scenarios provide two complementary levels of comparison: (i) the first 12 cases quantify the coupled influence of transverse repair spacing and patch architecture, whereas (ii) Cases 13–15 isolate the effect of repair orientation and in-line spacing using a common patch architecture. The complete set of configurations and their corresponding geometric and repair parameters are summarized in Table 4.

**Figure 10:**
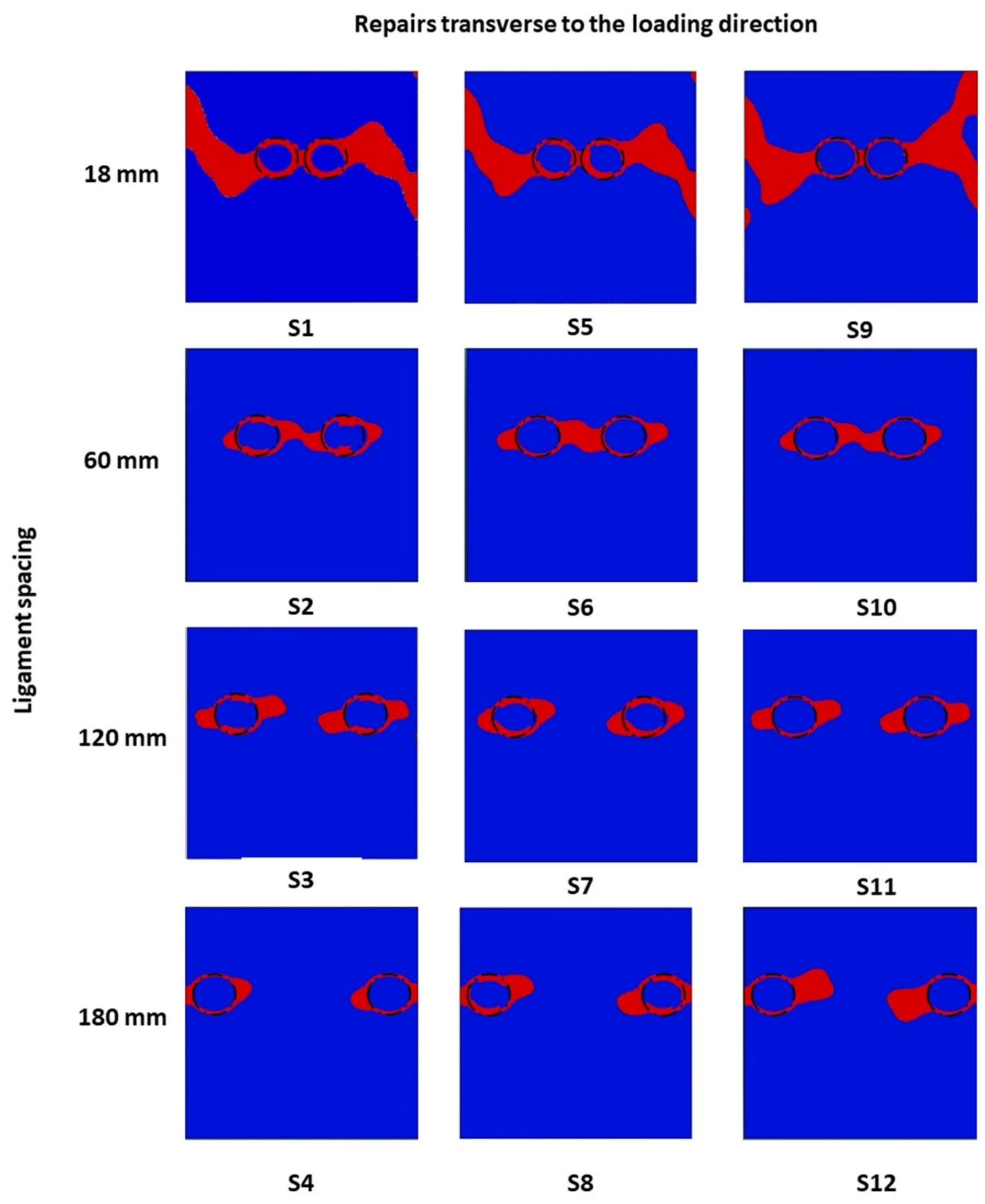
Interfacial damage variable, CSDMG, at the onset of final failure for the twelve configurations with the two repairs arranged transverse to the loading direction. Rows correspond to ligament spacings of 18, 60, 120, and 180 mm, while columns correspond to the original, matched, and angled patch lay-up strategies (from left to right). Red denotes complete decohesion of the bond line (CSDMG = 1), whereas blue denotes an intact interface. Black dashed lines indicate the outlines of the repairs. All panels use identical contour limits, viewing direction, and scale, with each configuration shown at its respective failure load. Scenario identifiers are given below each panel.

**Figure 11:**
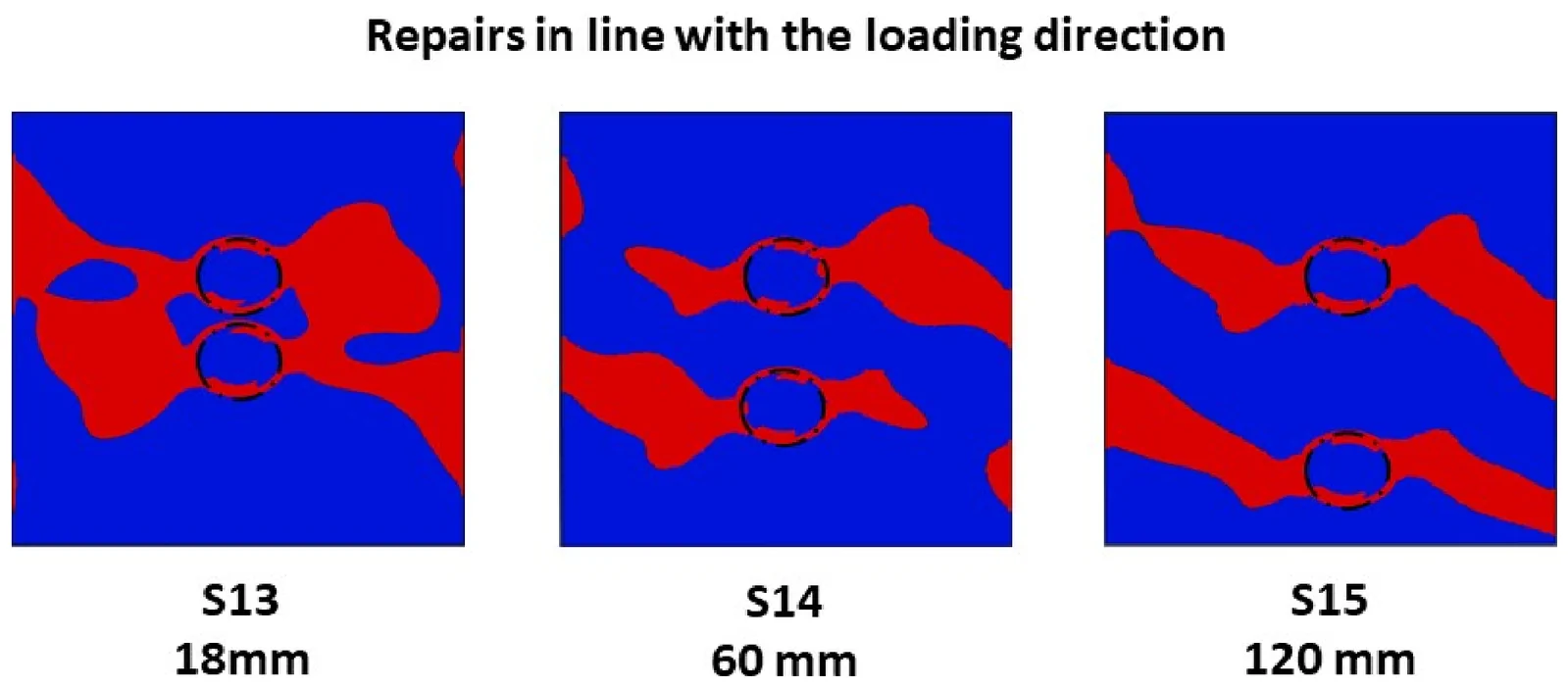
Interfacial damage variable, CSDMG, at the onset of final failure for the three configurations with the two repairs arranged in line with the loading direction, with ligament spacings of 18 mm, 60 mm, and 120 mm. Black dashed lines indicate the outlines of the repairs. The contour limits, viewing direction, and scale are identical to those used in Figure 10.

Figure 10 shows the interfacial damage state at the onset of final failure for the twelve transverse configurations (Scenarios 1–12), while Figure 11 presents the three in-line configurations (Scenarios 13–15). In Figure 10 and Figure 11, the red color denotes complete decohesion of the bond line. CSDMG is the scalar damage variable of the cohesive interaction defined in Equation (12); it is evaluated on the bonded surfaces between the repair plies and the stepped parent laminate of each repair, and the contours show these surfaces in plan view at their actual positions within the panel. The damage topology evolves in three stages with increasing ligament spacing. At 18 mm, a continuous band of complete decohesion connects the two bond lines across the ligament and extends to both free edges, eliminating a continuous load path across the panel width. At 60 mm, the two annular debonded regions remain connected through a narrow bridge along the line joining the repair centres. At 120 and 180 mm, the damage regions become fully separated, with each debonded zone confined primarily to the perimeter of its respective repair.

The transition from a coalesced to a separated damage field therefore occurs between 60 and 120 mm, coinciding with the spacing at which the maximum strength retention is obtained. At 180 mm, although the two damage fields remain separated, strength retention decreases. In this configuration, the debonded regions are positioned closer to the panel free edges, indicating that repair-to-edge interaction becomes more influential as the inter-repair spacing increases.

The effect of patch lay-up on damage topology is less pronounced than that of ligament spacing. At each spacing, the original, stiffness-matched, and angled lay-ups exhibit similar overall damage patterns, while the lay-up primarily affects the extent of the debonded regions rather than the spacing at which damage coalescence ceases.

The in-line configurations in Figure 11 exhibit the largest debonded areas among the investigated cases, with relatively little variation between 18 and 120 mm spacing. The debonding bands develop obliquely, and the two repairs are located on different transverse planes; consequently, no single transverse section is completely interrupted by the damage. Despite the greater extent of interfacial decohesion, these configurations retain higher residual strength than the transverse configurations at all spacings except 120 mm, demonstrating that the spatial distribution of damage is more influential than the debonded area alone.

#### 3.3.1. Effect of Ligament Spacing on Load Redistribution

**Figure 12:**
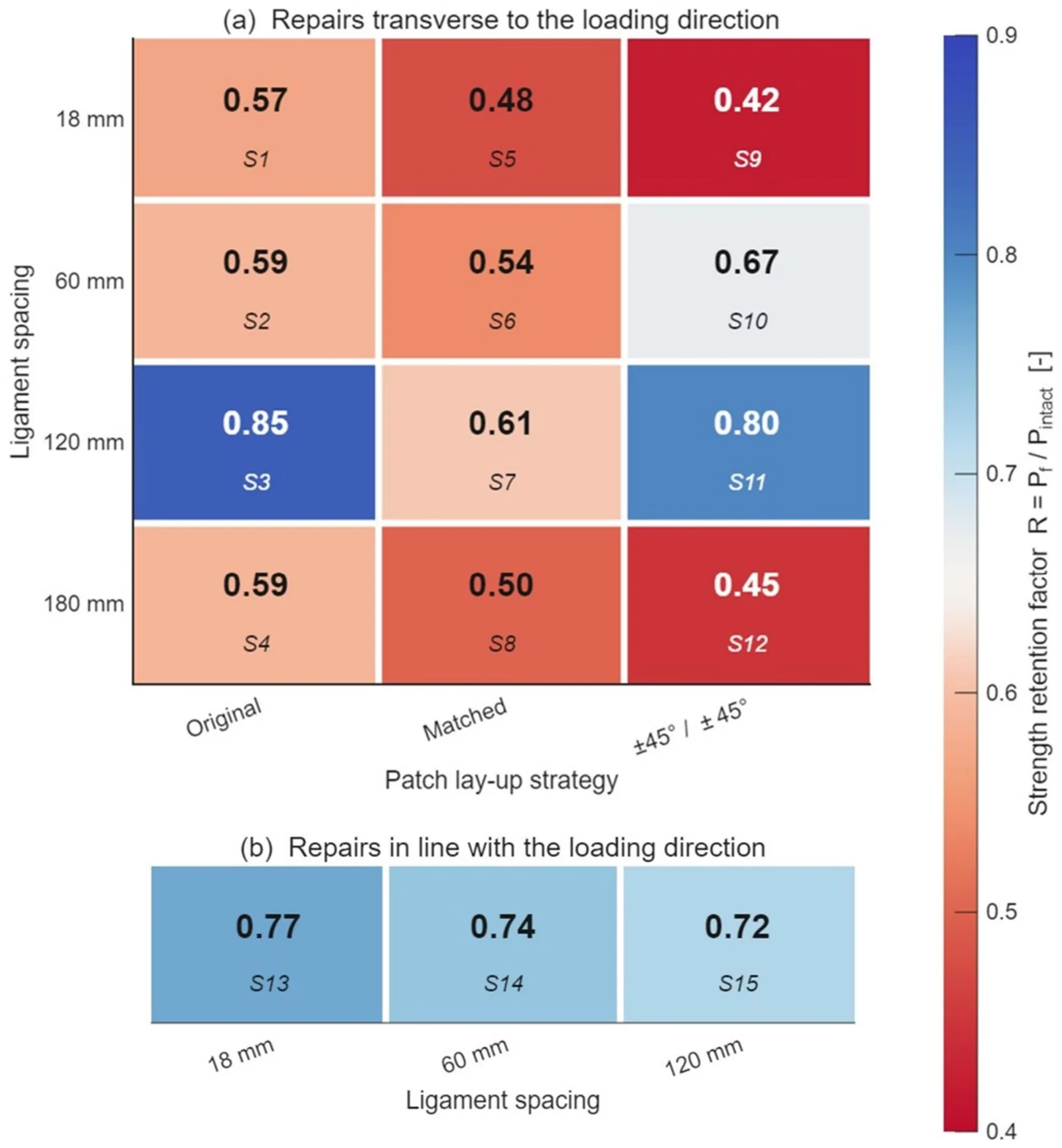
Strength retention factor, (R =$P_{f}/P_{intact}$), for the dual-repair panels, where =216 kN corresponds to the intact laminate strength of 600 MPa for a (150 × 2.4) mm section. (**a**) Transverse repair arrangements for four ligament spacings and three patch lay-up strategies. (**b**) In-line repair arrangements. Scenario identifiers are given below each value.

**Figure 13:**
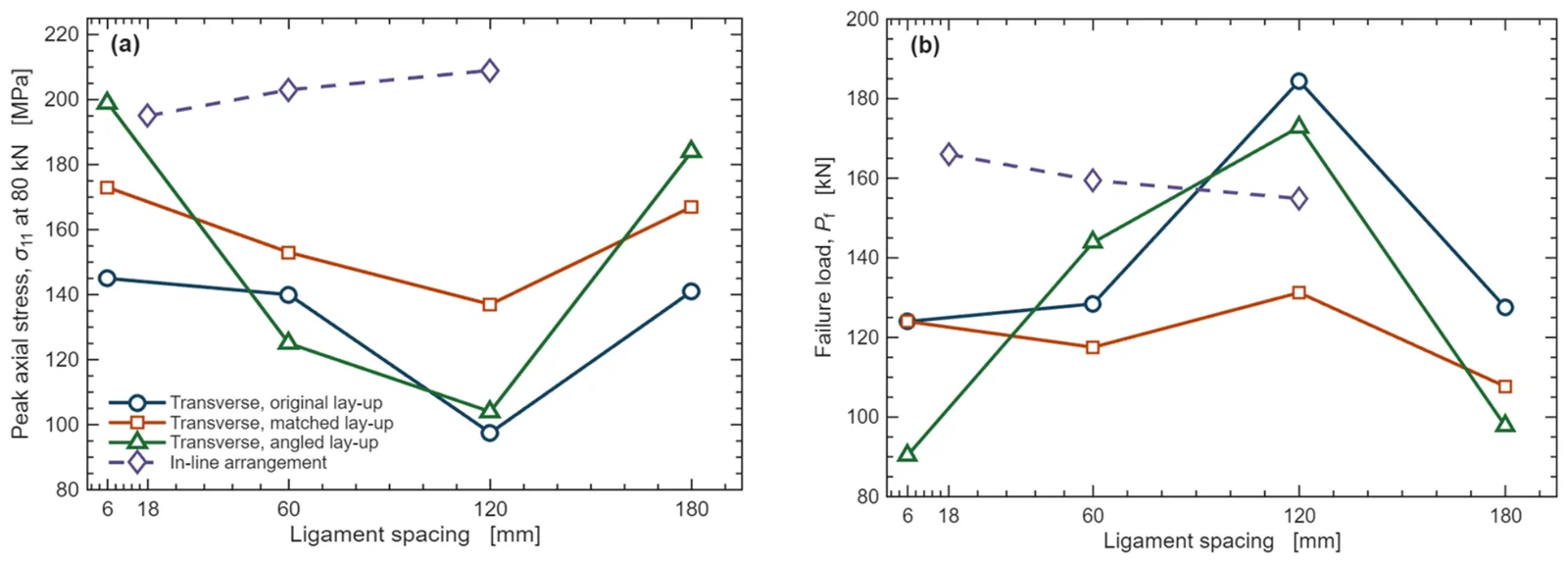
Effect of ligament spacing, patch lay-up strategy, and repair arrangement on the response of dual-repair panels. (**a**) Peak axial stress, (S11), at an applied load of 100 kN, corresponding to the linear-elastic regime before cohesive damage initiation. (**b**) Failure load. Scenarios 1–12 represent transverse repair arrangements with ligament spacings of 18, 60, 120, and 180 mm, whereas Scenarios 13–15 represent in-line arrangements with spacings of 18, 60, and 120 mm.

The strength retention factors of the dual-repair panels are summarized in Figure 12, and the peak axial stress and failure load ($P_{f}$) for the twelve configurations are compared in Figure 13. For the comparison strength retention factor R is calculated (R = $P_{f}/P_{intact}$). Since existing Structural Repair Manuals do not define a general spacing criterion for multiple composite repairs, ligament distances from 18 to 180 mm were investigated. The transverse configurations exhibit a non-monotonic response. For the original lay-up, the peak stress decreases from 145 MPa at 18 mm to 97.5 MPa at 120 mm, while the failure load increases from 124 to 184 kN. The high stress at small spacing is associated with the interaction of the stress fields generated by the adjacent repairs. Beyond 120 mm, the trend reverses; at 180 mm, the peak stress increases to 141 MPa and the failure load decreases to 128 kN. This reversal is attributed to increasing interaction between the repair regions and the panel free edges. Accordingly, 120 mm provides the most favorable spacing for the transverse configuration. The in-line configurations are considerably less sensitive to spacing. Between 18 and 120 mm, the peak stress varies only from 195 to 209 MPa, with little change in failure load. This behavior indicates that spacing is a governing parameter primarily for transverse rather than in-line repair arrangements. A similar dependence on alignment relative to the load has been reported for laminates with multiple open holes [33].

In interpreting these results, it should be noted that the peak axial stress is a local quantity, evaluated at a fixed load of 100 kN in the linear-elastic regime (Figure 13a). The failure load and strength retention factor (R), in contrast, are global measures of the residual load-carrying capacity, and the conclusions of this study are based on these measures. The non-monotonic response can be explained by the load paths around the repairs. Each repair perturbs the load path in its vicinity: load is diverted around the cavity and the patch, and the axial stress in the adjacent parent laminate is raised over a distance comparable to the repair diameter, as for an open hole. For the closest spacing (L = 18 mm, i.e., 0.3 times the cavity diameter D = 60 mm), the two perturbed zones overlap in the ligament, which must carry the load diverted from both repairs; the interfacial damage of the two repairs coalesces across the ligament (Figure 10), and the effective load-bearing width is reduced. As L increases to 2D (120 mm), the perturbed zones separate and the ligament stress approaches the far-field value. At the same time, however, the repairs move towards the free edges: the distance between the outer repair ply and the free edge decreases from 85 mm (1.4D) for L = 18 mm to 34 mm (0.57D) for L = 120 mm and 4 mm (0.07D) for L = 180 mm. Because a free edge carries no transverse stress and offers no material through which the diverted load can bypass the repair, the narrow strip between the repair and the edge becomes the critical load path, and this repair-to-free-edge interaction causes the strength reduction at L = 180 mm. The favorable spacing thus corresponds to the configuration that balances the two load paths: the ligament between the repairs and the strips between the repairs and the free edges. In non-dimensional terms, this occurs for L ≈ 2D with an edge distance of about 0.6D in the present panel. For other panel dimensions, the favorable spacing is therefore expected to be governed by these ratios rather than by the absolute value of 120 mm. In a wider panel, or in a panel with supported edges, the edge interaction would set in at larger spacings, so that the strength would be expected to level off rather than decrease beyond L ≈ 2D. Because these ratios are derived from a single panel width and repair size, they should be regarded as indicative and should be confirmed for other geometries.

#### 3.3.2. Influence of Patch Stacking Sequence

The strength retention factors are presented in Figure 12. The ±45° angle-ply configuration exhibits the greatest sensitivity to spacing, with (R) increasing from 0.42 at 18 mm to 0.80 at 120 mm. The original lay-up varies from 0.57 to 0.85, whereas the stiffness-matched lay-up ranges from 0.48 to 0.61. These differences arise from the stiffness distribution within the repaired region. The original lay-up maintains the parent-ply orientations and therefore provides a more continuous load-transfer path, whereas the compliant ±45° lay-up introduces a substantial reduction in longitudinal stiffness. This effect is particularly detrimental at small ligament distances. The stiffness-matched configuration shows the lowest sensitivity to spacing but also the lowest retention at 120 mm. This result appears counterintuitive, since a patch whose overall in-plane stiffness matches that of the parent laminate would be expected to provide the most uniform load transfer. It can be explained by the load-transfer mechanism of a stepped-lap repair. Load is not transferred between the parent and repair plies through their butt-ended terminations, but by interfacial shear over the overlap of each step, where a repair ply is bonded onto the exposed surface of a parent ply. Efficient transfer therefore requires the repair ply and the parent ply that it overlaps at a given step to have the same orientation, so that their axial and shear stiffnesses are compatible across the bonded interface. In the stiffness-matched lay-up (Figure 8a), the orientations are matched level by level, i.e., each repair ply has the orientation of the parent ply at the same through-thickness position. Because of the stepped geometry, however, each repair ply overlaps a parent ply of a different level and therefore of a different orientation (0/90° onto ±45°, or vice versa): the global stiffness of the patch is matched, but the stiffness at each bonded overlap is not. In the baseline lay-up (Figure 8b), each repair ply has the same orientation as the parent ply onto which it is bonded at its step. The orientation mismatch at the overlaps of the stiffness-matched patch is expected to concentrate the interfacial shear stresses at the step ends and to promote decohesion within each repair at a lower load. Because failure is then governed by the bonded interfaces of the individual repairs rather than by the interaction between the two repairs, the stiffness-matched configuration is the least sensitive to ligament spacing, but it also yields the lowest retention at the favorable spacing of 120 mm. These results indicate that, for stepped-lap repairs, matching the ply orientation at each bonded overlap is more important than matching the global stiffness of the patch.

The three lay-ups can be interpreted through a common chain of mechanisms. The patch stiffness at the bonded overlaps determines the share of the load carried by the repair; the remaining load bypasses the repair through the surrounding parent laminate; and the load transferred into the patch is carried by interfacial shear, whose concentration at the step ends controls the onset of decohesion. In the baseline lay-up, the orientation of each repair ply matches that of the parent ply onto which it is bonded, so the patch attracts a load share close to that of the removed material and the interfacial shear is distributed over the steps; both the bypass load and the interfacial demand are moderate, and the highest retention is obtained (R = 0.85 at 120 mm). The compliant ±45° patch has a much lower longitudinal stiffness and therefore attracts less load, so more load bypasses the repair and the ligament stress increases. This is most detrimental when the bypass flows of two repairs converge in a narrow ligament (R = 0.42 at 18 mm), whereas at 120 mm, where the perturbed zones no longer overlap, the retention (R = 0.80) approaches that of the baseline lay-up. In the stiffness-matched lay-up, the global stiffness is matched, but the orientation mismatch at the overlaps raises the interfacial shear demand, so that failure is governed by decohesion within each repair rather than by the ligament. The lay-up thus controls the global residual strength in two ways: through the bypass load, which couples the lay-up to the ligament spacing, and through the interfacial shear demand, which limits the retention largely independently of spacing.

Overall, ligament spacing and patch lay-up are coupled design parameters. The original lay-up provides the highest retained strength at the optimum transverse spacing, whereas the compliant ±45° configuration is least favorable at small spacing. The in-line configurations retain R = 0.72–0.77 despite exhibiting the highest peak stresses. This confirms that local peak stress alone does not directly represent global load-carrying capacity, as the transverse arrangement is more strongly governed by the reduced effective ligament.

The parametric results are obtained from deterministic analyses with nominal material properties, and a statistical treatment was therefore not performed. The robustness of the conclusions can nevertheless be assessed against the sources of uncertainty quantified in Section 2.3.1 and Section 3.1. The discretization error is below 2.5% of the peak load for element sizes of 0.1 mm and below. The validation shows a uniform bias of −6.1% to −6.4%, nearly identical for configurations with different failure mechanisms, while the strength ratios between configurations are reproduced within 0.5%. The experimental scatter of the reference tests corresponds to coefficients of variation of 2.0–4.0% (Table 3). By comparison, the differences in R that support the main conclusions are considerably larger: 0.28 between the closest and the most favorable transverse spacing of the baseline patch (0.57 versus 0.85), 0.24 between the baseline and stiffness-matched patches at 120 mm (0.85 versus 0.61), and 0.38 between the extreme spacings of the ±45° patch (0.42 versus 0.80). Since these differences exceed the combined discretization, modelling and experimental uncertainties by a wide margin, the ranking of the configurations and the identified trends are considered robust, whereas the absolute values of R should be interpreted with an uncertainty of the order of the validation bias.

## 4. Conclusions

This study investigated the coupled effects of ligament spacing, patch lay-up, and repair arrangement on the residual strength of dual-site stepped-lap repaired CFRP panels. The conclusions below are drawn from finite element simulations of a 400 × 320 mm panel under uniaxial tension and apply to the configurations analyzed; their potential application to repair design and the limitations of the framework are addressed at the end of this section. The results demonstrate that the effect of ligament spacing is strongly dependent on repair orientation. For transverse arrangements, the response was distinctly non-monotonic: strength retention increased from R = 0.57 at 18 mm ligament spacing to a maximum of R = 0.85 at 120 mm ligament spacing for the original lay-up, before decreasing to R = 0.59 at the ligament spacing of 180 mm. This behavior reflects the competing effects of repair-to-repair interaction at small spacing and repair-to-free-edge interaction at large spacing.

Patch lay-up further influences the sensitivity to ligament spacing. The compliant ±45° lay-up exhibited the greatest variation in strength retention, with R ranging from 0.42 to 0.80, whereas the stiffness-matched lay-up showed a narrower range from 0.48 to 0.61. In contrast, the in-line configurations exhibited limited sensitivity to spacing, retaining between 0.72 and 0.77 of the intact strength despite relatively high local peak stresses. This demonstrates that local stress concentration alone does not fully characterize the global residual strength of multi-site repairs.

Overall, the findings indicate that repair orientation relative to the principal load path is a primary governing parameter, while ligament spacing and patch stiffness act as coupled design variables. The results provide a panel-level basis for assessing closely spaced bonded repairs and indicate that repair-layout criteria should account for both repair orientation and boundary proximity rather than relying on separation distance alone. These findings can support more structurally efficient wet lay-up repair strategies for multi-site composite damage.

Regarding aerospace applications, the modelling framework is built on aerospace-grade materials and practice: the M21/AS4C parent laminate, the G0904/HYSOL EA 9396 wet lay-up repair system and the stepped-lap geometry are representative of field repairs to composite airframe skins, and the shell-based formulation corresponds to the idealization used in the stress analysis of aircraft panels. The framework can therefore be used for the preliminary assessment and comparison of repair layouts for multi-site damage in aerospace composite panels, for example, to decide whether two adjacent damage sites should be repaired separately or merged into a single repair. It is not, however, a substitute for certification. The substantiation of a specific repair additionally requires consideration of the actual boundary conditions and of combined, shear and compressive loading including buckling; of fatigue and environmental (hot–wet) effects on the wet lay-up bond line; and experimental validation of dual-repair configurations within the building-block approach required by the airworthiness regulations [23,25,26].

The present findings should not be generalized beyond the following limitations of the modelling framework: (i) the dual-repair configurations have not been validated experimentally, and only the single-repair coupons were compared with test data; (ii) a single panel width, repair geometry and material system were analyzed; (iii) only quasi-static uniaxial tension with free longitudinal edges was considered; (iv) the conventional shell formulation does not capture delamination within the parent laminate or the patch; (v) nominal, deterministic material properties were used, and porosity was represented only through effective properties; and (vi) fatigue, environmental and impact effects were not considered.

## Figures and Tables

**Table 1:** Material properties of the M21/AS4C [11,12] and Hexforce G0904 using HYSOL EA 9396 adhesive [11,37].

Property	Symbol	Hexforce G0904	M21/AS4C
Elastic Modulus (GPa)	E11	49.6	61.0
	E22	49.6	61.0
	E33	8.0	8.9
Shear Modulus (GPa)	G12	3.3	4.2
	G13, G23	2.8	3.8
Tensile Strength (MPa)	XT	517	930
	YT	517	940
Compression Strength (MPa)	XC	483	883
	YC	483	883
Shear Strength (MPa)	S12	60	96
	S13, S23	34	64
Poisson ratio	ν12	0.045	0.05
	ν13, ν23	0.28	0.3

**Table 2:** Mechanical properties of the HYSOL EA 9396 adhesive [11,12].

Property	Symbol	HYSOL EA 9396
Elastic Modulus (GPa)	*E*	2.7
Shear Modulus (GPa)	*G*	0.7
Tensile Strength (MPa)	$t_{n}^{0}$	55
Shear Strength (MPa)	$t_{s}^{0}$, $t_{t}^{0}$	26
Tensile Stiffness (N/${\mathrm{mm}}^{3}$)	*K*_n_	$10^{6}$
Shear Stiffness (N/${\mathrm{mm}}^{3}$)	*K*_s_, *K*_t_	$10^{6}$
Mode-1 Fracture Energy (N/mm)	*G*_IC_	0.4
Mode-2,3 Fracture Energy (N/mm)	*G*_IIC_, *G*_IIIC_	0.7

**Table 3:** Comparison of the predicted tensile strengths with the experimental results of [12]. Predicted strengths are net-section values obtained by dividing the maximum reaction force at the loaded end by the initial cross-sectional area of 150 mm × 2.4 mm. Deviations are computed with respect to the average measured strength, and the recovery rate is defined with respect to the intact laminate strength of 600 MPa.

Configuration	Experimental Average (MPa)	Experimental Maximum (MPa)	CoV (%)	Max. Reaction Force (kN)	Present FE Model (MPa)	Deviation (%)	Experimental Recovery (%)	Present FE Model Recovery (%)
Damaged	244	255	3.0	82.2	228.3	−6.4	40.0	38.1
Stepped	149	152	2.5	50.2	139.5	−6.4	24.8	23.3
Wet lay-up repaired	398	416	4.0	134.6	373.9	−6.1	66.0	62.3
Intact	600	614	2.0	—	—	—	—	—

**Table 4:** Definition of the multi-site repair scenarios investigated in the panel-level parametric study.

Scenario	Repair Arrangement	Ligament Distance, L (mm)	Patch Architecture	Purpose
1	Transverse	18	Baseline	Effect of close repair-site interaction
2	Transverse	60	Baseline	Effect of transverse spacing
3	Transverse	120	Baseline	Effect of transverse spacing
4	Transverse	180	Baseline	Near-independent repair response
5	Transverse	18	Stiffness-matched	Effect of patch stiffness at close spacing
6	Transverse	60	Stiffness-matched	Effect of patch stiffness and spacing
7	Transverse	120	Stiffness-matched	Effect of patch stiffness and spacing
8	Transverse	180	Stiffness-matched	Near-independent repair response
9	Transverse	18	Compliant [±45°/±45°]	Effect of compliant patch at close spacing
10	Transverse	60	Compliant [±45°/±45°]	Effect of patch compliance and spacing
11	Transverse	120	Compliant [±45°/±45°]	Effect of patch compliance and spacing
12	Transverse	180	Compliant [±45°/±45°]	Near-independent repair response
13	In-line	18	Baseline	Effect of close in-line interaction
14	In-line	60	Baseline	Effect of in-line spacing
15	In-line	120	Baseline	Effect of in-line spacing

## Data Availability

Data is contained within the article.
